# Complementary phase responses via functional differentiation of dual negative feedback loops

**DOI:** 10.1371/journal.pcbi.1008774

**Published:** 2021-03-08

**Authors:** Koichiro Uriu, Hajime Tei

**Affiliations:** Graduate School of Natural Science and Technology, Kanazawa University, Kanazawa, Japan; King’s College London, UNITED KINGDOM

## Abstract

Multiple feedback loops are often found in gene regulations for various cellular functions. In mammalian circadian clocks, oscillations of *Period1* (*Per1*) and *Period2* (*Per2*) expression are caused by interacting negative feedback loops (NFLs) whose protein products with similar molecular functions repress each other. However, *Per1* expression peaks earlier than *Per2* in the pacemaker tissue, raising the question of whether the peak time difference reflects their different dynamical functions. Here, we address this question by analyzing phase responses of the circadian clock caused by light-induced transcription of both *Per1* and *Per2* mRNAs. Through mathematical analyses of dual NFLs, we show that phase advance is mainly driven by light inputs to the repressor with an earlier expression peak as *Per1*, whereas phase delay is driven by the other repressor with a later peak as *Per2*. Due to the complementary contributions to phase responses, the ratio of light-induced transcription rates between *Per1* and *Per2* determines the magnitude and direction of phase shifts at each time of day. Specifically, stronger *Per1* light induction than *Per2* results in a phase response curve (PRC) with a larger phase advance zone than delay zone as observed in rats and hamsters, whereas stronger *Per2* induction causes a larger delay zone as observed in mice. Furthermore, the ratio of light-induced transcription rates required for entrainment is determined by the relation between the circadian and light-dark periods. Namely, if the autonomous period of a circadian clock is longer than the light-dark period, a larger light-induced transcription rate of *Per1* than *Per2* is required for entrainment, and vice versa. In short, the time difference between *Per1* and *Per2* expression peaks can differentiate their dynamical functions. The resultant complementary contributions to phase responses can determine entrainability of the circadian clock to the light-dark cycle.

## Author summary

Gene regulatory networks underlie various cellular functions and often include multiple feedback regulations. Multiple feedback loops confer robustness on cellular systems, but whether and how they can obtain different functions is unclear. To address this question, we analyze the phase responses of mammalian circadian rhythms to light signals. In mammals, dual negative feedback loops (NFLs) of *Period1* (*Per1*) and *Period2* (*Per2*) genes are responsible for rhythm generation. Light signals induce transcription of both *Pers* mRNAs, shifting the phase of clocks. We show that the time difference between expression peaks of two repressors, as in expression of *Per* genes, leads to functional differentiation: the NFL with an earlier expression peak of repressor, as *Per1*, contributes mainly to advancing the clock, whereas the other NFL with a later peak of repressor, as *Per2*, contributes to delaying the clock. These complementary contributions suggest that the ratio of light-induced transcription rates between two *Per* genes underlies the differences in phase responses between different mammalian species. Furthermore, the relation between the circadian and light-dark periods determines the ratio of light-induced transcription rates required for entrainment. Our study reveals a mechanism for functional differentiation of dual NFLs and its significance in circadian clock systems.

## Introduction

Complex gene regulatory networks are responsible for diverse cellular functions, such as transcriptional switches, adaptation, noise filtering, and genetic oscillations [1–3]. These gene regulatory networks often include multiple feedback regulations. Naturally, redundancy in multiple feedback regulations confers robustness on the system, but understanding whether and how redundant feedbacks acquire different functions is less resolved. Here we address this question with the interacting negative feedback loops (NFLs) in the mammalian circadian clock system.

Almost all known organisms possess circadian clocks that can set a subjective time for an individual in a constant environment and regulate behavioral and physiological rhythms. A fundamental characteristic of these circadian clocks is their ability to entrain to zeitgebers, including light-dark (LD) cycles. Entrainment properties of circadian clocks have been studied by measuring responses to brief light signals under conditions of constant darkness. In mammals including human, a light signal administered at subjective dawn advances the clock, whereas one at subjective dusk or night delays the clock [4,5]. Such advance and delay of the circadian clock are termed phase shifts. Furthermore, plotting phase shifts as a function of the time of light administration results in a phase response curve (PRC), which predicts how the circadian clock entrains to LD cycles [4,6,7].

**Fig 1:**
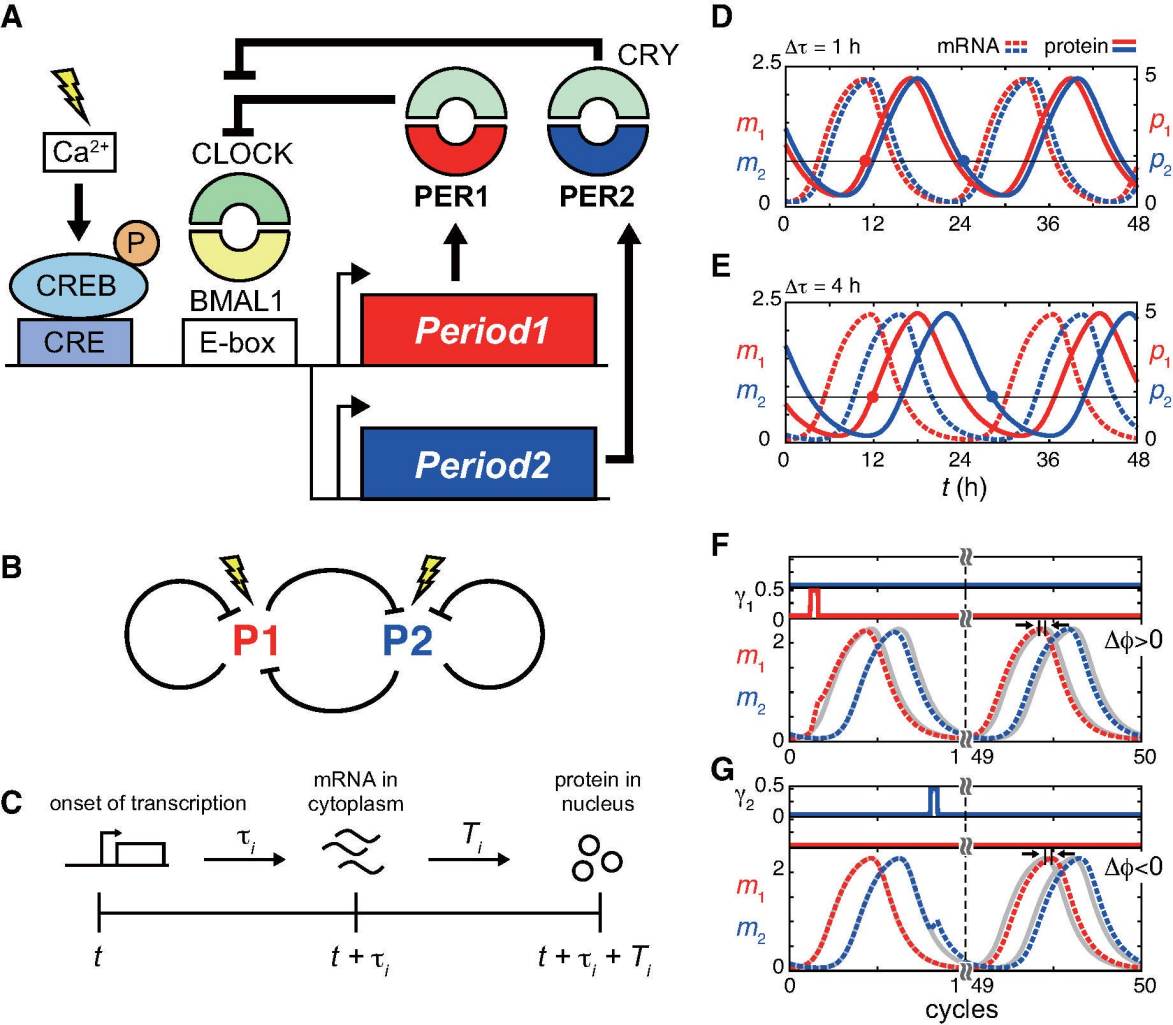
Rhythms generated by two interacting negative feedback loops. (A) Gene regulatory network of mammalian *Period1* (*Per1*) and *Period2* (*Per2*). The transcription of *Per1* and *Per2* mRNAs is induced by the CLOCK-BMAL1 complex through E-box in the promoter. PER1 and PER2 proteins form a complex with CRYPTOCHROME (CRY) and repress their own transcription. Light signals increase intracellular Ca^2+^ concentration and activate the cAMP response element binding protein (CREB) by phosphorylation, which binds to the CRE element in the promoters of *Per* genes and induces transcription of *Per* mRNAs. (B) Schematic of two interacting negative feedback loops. (C) Time delays in mRNA and protein production. (D), (E) Time series of repressor mRNAs (*m*_1_ and *m*_2_) and proteins (*p*_1_ and *p*_2_) with (D) Δ*τ* = 1 h, and (E) Δ*τ* = 4 h in the absence of light signals. Red circles indicate the time at which *p*_1_ exceeds the dissociation constant *K* of the promoters. Blue circles indicate the time at which *p*_2_ becomes smaller than *K*. (F), (G) Light-induced phase shifts with (F) only *P1* induction ϵ_1_ = 0.5 and ϵ_2_ = 0, and (G) only *P2* induction ϵ_1_ = 0 and ϵ_2_ = 0.5 in Eq (2). Time series of *m*_1_ (red dotted line) and *m*_2_ (blue dotted line) with light administration are shown. Gray solid lines indicate the time series of *m*_1_ and *m*_2_ without light administration. The phase shift Δ*ϕ* is measured as the peak time difference between these perturbed and unperturbed trajectories after 50 cycles. *P1* induction in (F) results in phase advance Δ*ϕ* > 0, whereas *P2* induction in (G) results in phase delay Δ*ϕ* < 0. Parameter values in Eqs (1) and (2) are described in S1 Text.

Zeitgebers, including light signals, shift the phase of the circadian clock by affecting its molecular machinery. Negative feedback regulations of circadian clock genes with time delays generate oscillations in their gene expression with a nearly 24-hour period. In mammals, the circadian clock genes *Period1* (*Per1*) and *Period2* (*Per2*) encode the transcriptional repressors of their own promoters (Fig 1A). Transcription of *Per1* and *Per2* mRNAs is induced by the CLOCK-BMAL1 complex through its binding to the E-box element (CACGTG) in their promoters. Each of the PER1 and PER2 proteins forms a complex with co-repressor CRYPTOCHROME1 or 2 (CRYs), then represses the transcriptional activity of CLOCK-BMAL1, closing an NFL. Also, since the molecular structures and functions of PER1 and PER2 proteins are similar [8,9], the repression of CLOCK-BMAL1 activity by PER1 and PER2 results in mutual repression between them (Fig 1A and 1B). The dual NFLs of *Per1* and *Per2* confer the regularity of gene expression rhythms, increasing the robustness of the circadian clock system [10,11]. Interestingly, one of the notable differences between *Per1* and *Per2* is the peak time of gene expression. In mice and rats, *Per1* expression peaks at subjective midday, and is followed by *Per2* expression, with about a 4-hour delay in the central pacemaker tissue, the suprachiasmatic nucleus (SCN) as indicated by *in-situ* hybridization [12–14] and q-PCR (e.g. ref. [15] and S1B Fig in ref. [16]) for the two *Per* genes. Moreover, in human U2OS cells, 2-hour difference between the expression peaks of *PER1* and *PER2* mRNAs was also observed [17]. Previous work has identified positive feedback of *Per2* as a possible mechanism for the 4-hour peak time difference [18]. In addition, a separate study indicated that a functional non-canonical E-box (CATGTG) in the *Per2* promoter also regulated the peak time of *Per2* expression [15]. However, it remains unclear whether the peak time difference between *Per1* and *Per2* reflects their different functions in entrainment to LD cycles, as well as the determination of period and amplitude of oscillation.

In addition to sustained rhythm generation, *Per1* and *Per2* also play key roles in phase responses of the mammalian circadian clock to light signals. Light signals induce the transcription of both *Per1* and *Per2* mRNAs in the SCN (Fig 1A) [12,19–23]. The resultant elevated levels of PER1 and PER2 proteins contribute to phase shifts of the clock and entrainment to LD cycles [7,24,25]. In addition to the difference in the peak times of *Per1* and *Per2* expression described above, experimental studies have shown that the magnitude of mRNA induction by light signals may also differ between them. For example, induction of *Per1* mRNA by a short light pulse was stronger than that of *Per2* in rat and hamster SCNs [12,26]. Yet, the biological significance of different induction levels between *Per1* and *Per2* by light signals for entrainment has also not been addressed.

Motivated by these experimental observations, here we use mathematical modeling and simulations to analyze the phase responses of two interacting NFLs to light signals. We show that a time difference between the expression peaks of the two repressors in the NFLs, as observed in *Per1* and *Per2*, leads to functional differentiation in their phase responses to light signals. Specifically, the repressor with an earlier peak time (i.e. *Per1*) mainly contributes to phase advance, whereas the repressor with a later peak time (i.e. *Per2*) contributes to phase delay. Due to these complementary contributions, the ratio of light-induced transcription rates between *Per1* and *Per2* determines the proportion of phase advance and delay zones in a PRC, and thereby determines the entrainability of a circadian clock to a 24-hour LD cycle. Thus, the dual NFLs can possess different dynamical roles according to the peak time difference, resulting in the tunability of phase responses to external input signals.

## Methods

### Time delay model for two interacting NFLs

To analyze phase responses of oscillations to light signals, we consider two interacting NFLs composed of transcriptional repressor genes *P1* and *P2*, corresponding to mammalian *Per1* and *Per2*, respectively (Fig 1B). Their protein products bind to each promoter and repress transcription in both. In general, transcription, translation, and transport of these protein products between the cytoplasm and nucleus require a certain time to be completed. Thus, the NFLs inherently include time delays, and such delayed negative feedbacks are known to produce self-sustained oscillations [1,27,28]. To describe the delayed feedbacks, we adopt a set of differential equations with time delay parameters [29–31]. The model includes *P1* and *P2* gene products, namely the levels of mRNAs *m*_*i*_ available for translation in the cytoplasm, and those of proteins *p*_*i*_ in the nucleus, as variables (*i* = 1,2). The time evolution of these mRNA and protein levels are described as:

$$\frac{dm_{i}(t)}{dt}=\frac{\beta }{1+{\left(p_{1}(t-\tau_{i})/K\right)}^{n}+{\left(p_{2}(t-\tau_{i})/K\right)}^{n}}+\gamma_{i}(t-{\bar{\tau }}_{i})-\alpha \cdot m_{i}(t),$$ (1a)

$$\frac{dp_{i}(t)}{dt}=v\cdot m_{i}(t-T_{i})-\mu \cdot p_{i}(t),$$ (1b)

where *β* is the maximum light-independent transcription rate, *α* is the degradation rate of mRNA, *v* is the translation rate, and *μ* is the degradation rate of protein. We assume the linear degradation of both mRNAs and proteins. The first term of Eq (1a) represents the regulation of transcription independent of light signals, such as through E-box. We describe transcriptional repression by the repressor proteins with a Hill function of the coefficient *n* and the dissociation constant *K* of the repressor proteins to the promoters. To obtain this Hill function, we assume the cooperative binding of repressor proteins where binding of a protein to one of the binding sites in the promoter makes the protein of same type more likely to bind to neighboring binding sites [29,32]. However, the competition for the promoter would not affect results presented below, because we introduce the peak time difference between *m*_1_ and *m*_2_ later, and resultant separate expression of proteins eventually relax the competition. *τ*_*i*_ in Eq (1a) represents time required for the processes to produce matured mRNAs *m*_*i*_ available for translation, such as splicing, modification, and transport from the nucleus to the cytoplasm (Fig 1C). Hence, the first term in Eq (1a), describing the rate of increase of the matured mRNAs at time *t*, is determined by the protein levels at the onset of transcription *t* − *τ*_*i*_, *p*_1_(*t* − *τ*_*i*_) and *p*_2_(*t* − *τ*_*i*_). Note that the timing of transcriptional repression by repressor proteins is the same for both *P1* and *P2* genes. For example, if the levels of P1 and P2 proteins become high enough to repress transcription at time *t*_*r*_, this effect of repression is reflected at time *t* = *t*_*r*_ + *τ*_1_ in *m*_1_, and at *t* = *t*_*r*_ + *τ*_2_ in *m*_2_. Similarly, *T*_*i*_ in Eq (1b) represents the time required to translate mRNA and transport the products into the nucleus (Fig 1C). In the mouse SCN, the time difference between the expression peaks of a *Per* mRNA and the corresponding PER protein was about 4-6 hours [33]. Based on this data, we set *T*_1_ = *T*_2_ = 5 h unless mentioned otherwise in this study.

With appropriate values of time delays and reaction parameters, the model generates a stable limit cycle (Fig 1D and 1E). Although the regularity of autonomous rhythms in *Per1* or *Per2* deficient SCN was impaired [10,11], for simplicity, we choose a parameter regime where each single NFL can sustain rhythms even in the absence of the other NFL. A previous study indicated that a positive feedback loop (PFL) of *Per2* was considered as a possible mechanism underlying the 4-hour peak time difference between *Per1* and *Per2* [18]. However, for simplicity, Eq (1) does not include a PFL. Instead, the peak time difference between *m*_1_ and *m*_2_ is controlled by the difference in time delays Δ*τ* = *τ*_2_ – *τ*_1_ in transcription through E-box in Eq (1a) (Fig 1D and 1E). In fact, another experimental study showed that the peak time of *Per2* expression was also regulated by a functional non-canonical E-box in the *Per2* promoter in mice [15]. Note that Δ*τ* and the peak time difference between *m*_1_ and *m*_2_ are equivalent in Eq (1) (S1A and S1B Fig). In addition, Δ*τ* also reflects the difference in phases measured by the first Fourie components of *m*_1_ and *m*_2_ (S1A and S1C Fig), validating Δ*τ* as a measure for phase difference. To focus on just the effect of the peak time difference between *P1* and *P2*, we first assume that the maximum light-independent transcription rate, translation rate, degradation rate, and dissociation constants are the same between the two NFLs in Eq (1). We examine the effects of differences in the values of reaction parameters between *P1* and *P2* later in the result section.

The function *γ*_*i*_ in Eq (1a) describes the transcription of mRNA induced by light signals. We assume the following rectangular function for this light-induced transcription [34]:

$$\gamma_{i}(t)=\{\begin{array}{l} \epsilon_{i}\;t_{l}\leq t\leq t_{l}+T_{d}\\ 0\;\text{otherwise}, \end{array}$$ (2)

where *t*_*l*_ is the time at which a light signal is administered and *T*_*d*_ is the duration of the light signal (Fig 1F and 1G). In this paper, *t*_*l*_ = 0 indicates light administration starting at the time of an *m*_1_ trough (Fig 1F and 1G). Since the expression levels of *Per1* mRNA start to increase at dawn in the mouse SCN [16,35], time around a *m*_1_ trough roughly corresponds to subjective dawn, and time 12-hour after corresponds to subjective dusk. A light signal induces the transcription of the repressor *P*_*i*_ at a rate *ϵ*_*i*_. ${\bar{\tau }}_{i}$ in Eq (1a) represents the time delay in mRNA production induced by light signals. We assume that ${\bar{\tau }}_{1}<{\bar{\tau }}_{2}$, because the levels of *Per1* mRNA are elevated quicker than that of *Per2* after light administration in the mouse SCN [9,36].

To compute the phase shift caused by the light signals, we simulate the time evolution of mRNAs and proteins with a light pulse at time *t*_*l*_ (perturbed) and those without a light pulse (unperturbed). We simulate 50 cycles after the administration of the light signal to stabilize the trajectory (Fig 1F and 1G). Then, we calculate the peak time difference Δ*ϕ* of *P1* mRNAs by subtracting the peak time of the perturbed trajectory from that of the unperturbed one. A positive value of Δ*ϕ* indicates phase advance due to the light signal (Fig 1F), whereas a negative value of Δ*ϕ* indicates phase delay (Fig 1G). Thus, we define Δ*ϕ* as the phase shift induced by the light signal. In later sections, we may denote the phase shift as a function of the light-induced transcription rates of *P1* and *P2*, as Δ*ϕ* = Δ*ϕ*(*ϵ*_1_, *ϵ*_2_).

In this study, we analyze the dependence of phase responses to light signals on the time delay parameters *τ*_*i*_ in Eq (1a). We examine phase shifts within the ranges of the time delays where the period of autonomous oscillation *T*_*p*_ nears 24 hours. Changes in the time delay parameters may also affect *T*_*p*_. Therefore, to better compare the magnitude of phase shifts with different parameter values, we scale the duration of a light signal *T*_*d*_ in Eq (2) with *T*_*p*_ as *T*_*d*_ → (*T*_*p*_/24)*T*_*d*_ [34]. The details of numerical simulations and values of parameters are described in S1 Text and S1 File.

To quantify the shape of a PRC, we measure the unsigned areas of its advance (Δ*ϕ* ≥ 0) and delay (Δ*ϕ* < 0) zones, which we refer to *A* and *D* (*A* ≥ 0, *D* ≥ 0), respectively. Then, we compute the fraction of *A* to the total area:

$$R=\frac{A}{A+D}.$$ (3)

If *R* is close to one (zero), the typical response of the NFLs to light signals is phase advance (delay). *R* should be a function of the light-induced transcription rates ϵ_*i*_ in Eq (2), *R* = *R*(*ϵ*_1_, *ϵ*_2_).

## Results

### Dependence of the period and amplitude on the two NFLs

We start the analysis by examining the difference in dynamical functions between two NFLs in the regulation of autonomous oscillations. In Fig 1E, we introduce a 4-hour peak time difference between *m*_1_ and *m*_2_ with Δ*τ* = *τ*_2_ – *τ*_1_ = 4 h based on experimental data on mammalian *Per1* and *Per2*. With this peak time difference, we observe that the time at which *m*_1_ starts to decrease is close to the time when *p*_1_ surpasses the value of the dissociation constant *K* in Eq (1a), and the decrease in *m*_2_ follows 4-hour later (Fig 1E). If *p*_2_/*p*_1_ < 1 due to the peak time difference between the two proteins, the light-independent transcription rate in Eq (1a) can be approximated as *β*/(1 + (*p*_1_/*K*)^*n*^(1+(*p*_2_/*p*_1_)^*n*^)) ≈ *β*/(1+(*p*_1_/*K*)^*n*^) where we omit time delay parameters for notational simplicity. In addition, when *p*_1_ = *K* with *p*_2_/*p*_1_< 1, the light-independent transcription rate decreases to nearly the half of its maximum *β*/2. We confirm this approximation by observing the timeseries of the light-independent transcription rate and the levels of the two proteins with Δ*τ* = 4 h (S2A–S2C Fig). Hence, the passage time at which *p*_1_ surpasses *K* determines the onset of a repressed state where the transcription rate is less than *β*/2. We change this passage time of *p*_1_ with respect to *K* by shifting the value of the time delay in protein translation *T*_1_ in Eq (1b) with the condition *τ*_1_ + *T*_1_ < *τ*_2_ + *T*_2_ (S2D–S2G Fig). As *T*_1_ increases, *p*_1_ exceeds *K* at a later time. This later passage of *p*_1_ for *K* prolongs the transcription of both *P1* and *P2* mRNAs (S2D–S2F Fig), increasing the peak values of *m*_*i*_ and *p*_*i*_. Thus, the amplitude of oscillation increases with the increase in *T*_1_ (S2L Fig). In contrast, the duration of transcription is less sensitive to *T*_2_ (S2H–S2J Fig). Correspondingly, the increase in *T*_2_ in Eq (1b)) only weakly affects the amplitude (S2M Fig).

We then examine the increase of mRNA. We notice that when *p*_2_ decreases to *p*_2_ = *K*, the light-independent transcription rate increases to nearly the half of its maximum *β*/2 (S2A–S2C Fig). At the time *p*_2_ = *K*, *p*_1_/*p*_2_ < 1 in the presence of peak time difference (Fig 1E). Then, the light-independent transcription rate can be approximated as *β*/(1 + (*p*_2_/*K*)^*n*^((*p*_1_/*p*_2_)^*n*^ + 1)) ≈ *β*/(1+(*p*_2_/*K*)^*n*^) = *β*/2 (S2A–S2C Fig). Therefore, the time at which *p*_2_ becomes smaller than *K* sets the onset of induction state where the transcription rate is larger than *β*/2. This onset of induction state is delayed by the increase in *T*_1_ because it extends the time interval of *p*_2_ increase as described above (S2F, S2G and S2N Fig). Thus, the increase in *T*_1_ lengthens the autonomous period as well as amplitude (S2L and S2N Fig). The increase in the time delay *T*_2_ for *P2* translation in Eq (1b) also lengthens the autonomous period, as *T*_2_ delays the time for *p*_2_ to become smaller than *K* (S2H–S2K and S2O Fig). In summary, in the presence of peak time difference, the repressor with an earlier peak time determines the onset of transcriptional repression, whereas the other repressor with the later peak determines the onset of mRNA transcription.

### Complementary contributions of two interacting NFLs to PRC

Next, to study contributions of each NFL to phase responses, we draw PRCs with the induction of either *P1* or *P2* mRNA by light signals (Fig 1F and 1G). For simplicity, we assume that time delays in light-induced transcription are the same as those for light-independent transcription ${\bar{\tau }}_{i}=\tau_{i}$ (*i* = 1, 2) in Eq (1a).

**Fig 2:**
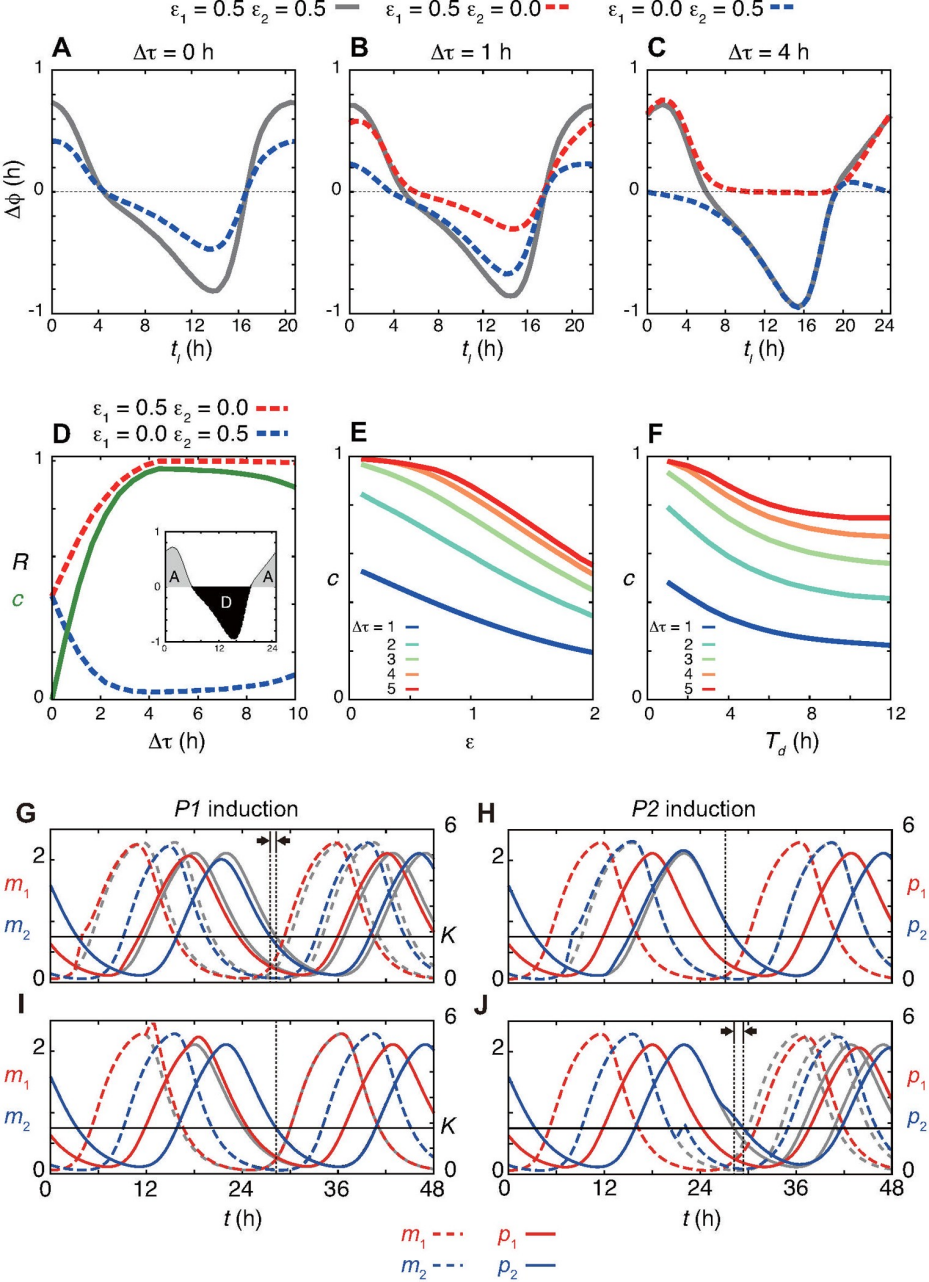
Complementary contributions to phase responses with time difference between expression peaks of two repressors Δ*τ*. (A)-(C) Phase response curves with (A) Δ*τ* = 0 h, (B) Δ*τ* = 1 h and (C) Δ*τ* = 4 h. Red and blue dotted lines indicate phase shifts caused by only *P1* or only *P2* induction, respectively. Gray solid lines indicate phase shifts caused by simultaneous induction of both *P1* and *P2*. In (A), red and blue lines overlap. (D) Dependence of area fraction *R* of advance zone *A* in Eq (3) and complementarity index *c* (green solid line) in Eq (4) on Δ*τ*. Red and blue dotted lines indicate *R* with only *P1* and only *P2* induction, respectively. Inset shows the areas of advance (gray) and delay (black) zones in a PRC as an example. (E), (F) Dependence of the index *c* on (E) the light-induced transcription rate *ϵ* and (F) the duration of induction *T*_*d*_ for different values of Δ*τ*. In (E), *c* is calculated with *R*(*ϵ*, 0) and *R*(0,*ϵ*). (G)-(J) Time series of *m*_*i*_ and *p*_*i*_ with light administration (red and blue lines). Gray dotted and solid lines indicate time series of *m*_*i*_ and *p*_*i*_, respectively, without light signals. Horizontal lines indicate the dissociation constant *K* of the promoters in Eq (1a). (G) *P1* or (H) *P2* induction when their mRNA levels are increasing. (I) *P1* or (J) *P2* induction when their mRNA levels are decreasing. Parameter values in Eqs (1) and (2) are described in S1 Text.

We first analyze the two identical NFLs Δ*τ* = *τ*_2_ – *τ*_1_ = 0 (Fig 2A). In this case, the waveforms of *P1* and *P2* mRNAs and proteins are the same. Correspondingly, PRCs obtained only by *P1* induction (ϵ_1_ = 0.5, ϵ_2_ = 0 in Eq (2)) are identical to those by *P2* induction (ϵ_1_ = 0, ϵ_2_ = 0.5) (Fig 2A). The PRC for simultaneous *P1* and *P2* inductions (ϵ_1_ = ϵ_2_ = 0.5) is approximately the sum of the phase shifts caused by each NFL, indicating the additivity of the two PRCs, Δ*ϕ*(*ϵ*_1,_*ϵ*_2_) ≈ Δ*ϕ*(*ϵ*_1_,0)+Δ*ϕ*(0,*ϵ*_2_). This additivity of PRCs probably originates from the additivity of phase sensitivity or infinitesimal PRCs in phase reduction theory [37–39].

Then, we introduce a peak time difference between *P1* and *P2* by increasing *τ*_2_. As the difference in time delays Δ*τ* becomes large, the difference in the contributions of *P1* and *P2* to PRCs increases (Fig 2B–2D). Notably, the induction of *P1* mRNA, which peaks earlier, contributes to phase advance rather than phase delay. The area of the advance zone *A* in the PRC, i.e. the area above zero phase shift, increases with Δ*τ* (Fig 2D). In contrast, the induction of *P2* mRNA contributes to phase delay as *R* decreases with Δ*τ* (Fig 2D). The PRC obtained by simultaneous induction of both *P1* and *P2* mRNAs is nearly the sum of each contribution, retaining the additivity of PRC (Fig 2B and 2C). Thus, the time difference between expression peaks of the two repressors separates their contributions to PRCs such that each NFL complements the other.

To quantify the magnitude of complementarity in the phase responses, we define an index:

c(ϵ)=R(ϵ,0)−R(0,ϵ), (4)

where *R*(*ϵ*, 0) is the area fraction of advance zone in a PRC obtained only by *P1* induction, with rate *ϵ* defined by Eq (3), and *R*(0, *ϵ*) is the area fraction obtained only by *P2* induction. *c* becomes large with Δτ as the contributions of two NFLs to phase shifts become complementary (Fig 2D). Interestingly, the index *c* increases until Δ*τ* ≈ 4, then it slowly decreases with Δ*τ* (Fig 2D), indicating the existence of optimal Δ*τ* for complementarity.

Subsequently, we examine the mechanism for this functional differentiation in phase responses (Figs 2G–2J and S3). P1 protein determines the time at which the light-independent transcription rate decreases to its half maximum as described above. If a light signal further induces transcription of *P1* mRNA when mRNA levels are increasing, P1 protein levels exceed the dissociation constant *K* in Eq (1a) earlier, due to increased translation by the excess mRNA (Fig 2G). Therefore, the light-independent transcription rate decreases to *β*/2 earlier (S3A Fig), which results in lower accumulation of both P1 and P2 proteins (Fig 2G). Consequently, the degradation of these proteins to levels lower than *K* takes less time, advancing the phase of oscillation. In contrast, light induction of *P1* mRNA at the time interval where its levels are decreasing does not affect the light-independent transcription, because P2 protein levels are still abundant (Figs 2I and S3C). At this time interval, conversely, the light induction of *P2* mRNA delays the time at which the light-independent transcription rate increases to *β*/2 by prolonging the transcriptional repression by excess P2 protein (Figs 2J and S3D). On the other hand, induction of *P2* mRNA when its levels are increasing only weakly advances the phase of oscillation (Figs 2H and S3B). This is because P1 protein is already abundant, masking the effect of *P2* induction. Note that these observed phase shifts are correlated with the transient change in the amplitude of P2 protein, like the covariation of the amplitude and period shown in the previous section.

The above analysis suggests that differences in time at which *p*_1_ and *p*_2_ become larger or smaller than the dissociation constant *K* are key to the complementary phase responses. These time differences can be influenced by not only Δ*τ* but also time delays in translation *T*_*i*_. Indeed, if *T*_2_ = *T*_1_ − Δ*τ*, the passage time at which *p*_2_ becomes smaller than *K* is the same as the corresponding passage time of *p*_1_ (S4A Fig). In this case, (*τ*_2_ + *T*_2_)/(*τ*_1_ + *T*_1_) = 1 and the index *c* is almost zero (S4D Fig). As we increase *T*_2_, this passage time of *p*_2_ with respect to *K* becomes later than that of *p*_1_ (S4B and S4C Fig) and *c* grows (S4D Fig). Similarly, as *T*_1_ becomes larger than *T*_2_, and closer to *T*_1_ = *T*_2_ + Δ*τ*, the value of index *c* decreases. Thus, the complementary phase responses require the condition (*τ*_2_ + *T*_2_)/(*τ*_1_ + *T*_1_) > 1. The experimentally observed 4-hour time difference between expression peaks of *Per1* and *Per2* mRNAs with similar time delays in translation *T*_1_ ≈ *T*_2_ is a way to satisfy this condition.

Next, we examine the dependence of these complementary phase responses on the parameters involved in light induction of *P1* and *P2* mRNAs. We confirm that the complementary contributions of dual NFLs to PRCs are preserved with different values of time delays in light-induced transcription ${\bar{\tau }}_{i}$ in Eq (1a), supporting the robustness of the results (S5A–S5C Fig). In contrast, we find that the index *c* decreases with an increase in light-induced transcription rate ϵ (Fig 2E). A longer duration of the light signal *T*_*d*_ also decreases *c* (Fig 2F). As the values of these parameters increase, *P1* and *P2* inductions also cause substantial phase delay and advance, respectively (S5D–S5I Fig). A strong light induction of *P1* mRNA at its trough results in *p*_1_ larger than both the dissociation constant *K* and *p*_2_, delaying the phase of oscillation (S5J Fig). Similarly, by a strong light induction of *P2* mRNA at its trough timing, *p*_2_ exceeds *K* before *p*_1_, advancing the phase (S5K Fig). However, as long as the PRCs remain continuous and of type-1 in simulations, *c* never reaches zero (Figs 2E and 2F and S5D–S5I), indicating that this complementarity is an inherent property of the two interacting NFLs with the time difference between the expression peaks of the repressors.

### Dependence of complementary phase responses on reaction parameters

In the previous section, we have studied the phase responses of the interacting NFLs as described in Eq (1), in which only time delays differ between the two loops. Since *Per1* and *Per2* gene products have highly similar protein sequences [9], their other reaction parameter values (e.g. translation and degradation rates, and dissociation constants to promoters) are expected to be similar. In fact, both PER1 and PER2 proteins are incorporated into the repressor complex with CLOCK-BMAL1 and CRY1/2 [40], confirming their similar dissociation constants. In addition, the degradation of both PER1 and PER2 is regulated by Casein kinase 1ε/δ [41]. Hence, it is worth examining whether the similar reaction parameters of *P1* and *P2* facilitate their complementary contributions to PRCs more than different values would. To investigate this possibility, we extend Eq (1) to incorporate differences in parameter values between the two NFLs (S1 Text). Then, we introduce the ratios of each reaction parameter between the two loops by nondimensionalizing the extended version of Eq (1) (S1 Text). Subsequently, we study the dependence of the complementarity index *c* on these reaction parameter ratios, keeping Δ*τ* = 4 h (S6A–S6E Fig).

We find that the complementarity index *c* is maximized if the ratios of the reaction parameters are close to one (i.e. the reaction parameters are similar between the two loops) (S6A–S6E Fig). If the production rate of the P1 protein is above or its degradation rate is below that of P2 protein, *P1* induction by light signals tends to cause both phase advance and delay. In such cases, the peak value of the P1 protein becomes higher than that of P2 protein (S6F Fig). Consequently, *p*_1_ levels are reduced to less than *K* near the same time as *p*_2_ levels are. Hence, the passage time of *p*_1_ with respect to *K* shifts to later than that of *p*_2_ by the light-induced elevation of *p*_1_ levels, delaying the time at which the light-independent transcription rate increases to *β*/2. In other words, complementary contributions of phase responses by a peak time difference are more likely to occur if the amplitudes of mRNAs and proteins are similar between two NFLs. Additionally, Hill coefficients in both NFLs should be large for such complementary contributions (S6G and S6H Fig).

If we constrain the reaction parameters in the two NFLs to be identical, as in Eq (1), the nondimensional model has only two reaction parameters – the ratio of protein degradation rate to mRNA degradation rate *μ*/*α* and the ratio of effective protein production rate to the dissociation constant *vβ*/(*α*^2^*K*) (S1 Text). If the ratio *vβ*/(*α*^2^*K*) is close to 1 in S6I–S6K Fig, the system converges to a steady state and no oscillation occurs. As this ratio becomes large, a stable limit cycle emerges via a Hopf bifurcation, and light induction of either *P1* or *P2* mRNA results in a continuous type-1 PRC in simulations. However, if the ratio is further increased, the PRC shifts to type-0. Therefore, we calculate the complementarity index *c* within the region where the PRC shape is type-1. *c* depends on *vβ*/(*α*^2^*K*) nonmonotonically and the peak is located near the Hopf bifurcation point (S6I–S6K Fig). As *vβ*/(*α*^2^*K*) becomes larger, *P2* induction by light signals starts to cause phase advance. If a light induction of *P2* mRNA occurs at around the trough of *p*_2_ with a high value of *vβ*/(*α*^2^*K*), *p*_2_ levels are more likely to surpass *K* due to the light induction. The elevated *p*_2_ levels above *K* at such timing reduce the subsequent peak value of *p*_2_, leading to the earlier relief of transcriptional repression.

The complementary index *c* also depends on the ratio of degradation rate of protein to that of mRNA, *μ*/*α*. As *μ*/*α* becomes large, *c* peaks at a larger value of *vβ*/(*α*^2^*K*) and its peak value increases (S6I–S6K Fig). Rapid protein degradation prevents the accumulation of P1 and P2 proteins after the light induction. Hence, after the light induction of *P1* mRNA at its decrease, P1 protein levels remain lower than P2 protein levels. Similarly, P2 protein levels stay lower than *K* after the light induction of *P2* mRNA at its trough. In this way, fast protein degradation facilitates the functional differentiation of *P1* and *P2* in phase responses. A previous experimental study reported that the half-life of PER2 protein fused with a luciferase reporter (PER2::LUC) in the mouse SCN slices was about 1.9 hours [42]. The data for the half-lives of *Per1* and *Per2* mRNAs in the SCN is currently not available. However, in NIH3T3 cells, the half-life of *Per2* mRNA was 0.9 hours and in embryonic stem cells, it was 2.9 hours [30,43,44]. Using these available data, we estimate *μ*/*α* = 0.47 ~ 1.52. The complementary phase responses can be observed within this range of *μ*/*α* as shown in S6I–S6K Fig.

### Complementary phase responses in dual NFLs including multiple states of mRNA and protein

So far, we have described the dynamics of repressors in dual NFLs using delay differential equations for the ease of controlling their peak time difference. Another description of time delays in NFLs is to model different states of mRNA and protein explicitly as the Goodwin-type models [1,27,28,45–50]. To examine whether complementary phase responses occur regardless of the description of time delays, here we analyze models that include multiple states of mRNAs and proteins as separate variables in ordinary differential equations (ODEs; S1 Text and S7 and S8 Figs).

First, to show how the inclusion of multiple states of mRNA affects period, amplitude and phase responses, we consider a single NFL with one repressor (S7A Fig). The model describes multiple states of mRNA (*m*_11_, *m*_12_, …, *m*_1*u*_) and proteins (*p*_11_,*p*_12_, …, *p*_1*r*_) with ODEs. *m*_11_ is the levels of transcribed nascent mRNA in nucleus and *m*_1*u*_ is the levels of matured mRNA at cytoplasm available for translation. Similarly, *p*_11_ is the levels of translated nascent repressor protein in cytoplasm and *p*_1*r*_ is those of functional protein in nucleus. For simplicity, we assume linear chains of state transition from *m*_1*i* − 1_ to *m*_1*i*_, and *p*_1*i* − 1_ to *p*_1*i*_ with time constants *η* and *λ*, respectively (S7A Fig). The details of the model are described in S1 Text. As the time constant *η* for the state transition of mRNA increases, the period decreases, whereas the amplitude increases (S7B and S7C Fig). In contrast, both the period and amplitude of oscillation increase with the state number of mRNA *u* (S7E and S7F Fig). Thus, these two parameters determine the delays in the production of functional mRNA. PRCs of this single NFL include both phase advance and delay zones (S7D and S7G Fig).

Next, we consider two interacting NFLs based on the description described above (S1 Text and S8A Fig). We assume that the time constants of state transition and state numbers are different between *P1* and *P2* mRNAs. A larger state number together with a smaller time constant of *P2* mRNA than those of *P1* mRNA generates peak time difference between their functional mRNAs with nearly 4 hours (S8B–S8E Fig). We observe complementary phase responses of the dual NFLs to light signals in this description as well (S8F and S8G Fig): light-induced transcription of *P1* mRNA mainly causes phase advance, whereas that of *P2* mRNA causes phase delay. Therefore, we conclude that complementary phase responses to light signals occur in the presence of the peak time difference, regardless of the description of time delays in dual NFLs.

### Diversity of PRC shapes resulting from different ratios of light-induced transcription rates

It is known that PRC shapes differ among mammalian species. For example, PRCs of mice include a larger delay zone than advance zone, whereas those of rats and hamsters include a larger advance zone [4,51]. How does this diversity of PRC shapes arise? The complementary contributions of *Per1* and *Per2* to the PRC described above could be the reason for the different mammalian PRCs. To verify this prediction, we simulate phase shifts at various values of light-induced transcription rate of *P1*, *ϵ*_1_ in Eqs (1) and (2), with a fixed total rate ϵ_*t*_ = ϵ_1_ + ϵ_2_. Thus, that of *P2* is ϵ_*2*_ = ϵ_*t*_ − ϵ_1_. Hereafter, we use Eqs (1) and (2) with identical values of the reaction parameters between the two NFLs. We quantify the difference in PRC shapes by the area fraction of advance zone *R*(*ϵ*_1_,*ϵ*_*t*_ – *ϵ*_1_) defined in Eq (3).

**Fig 3:**
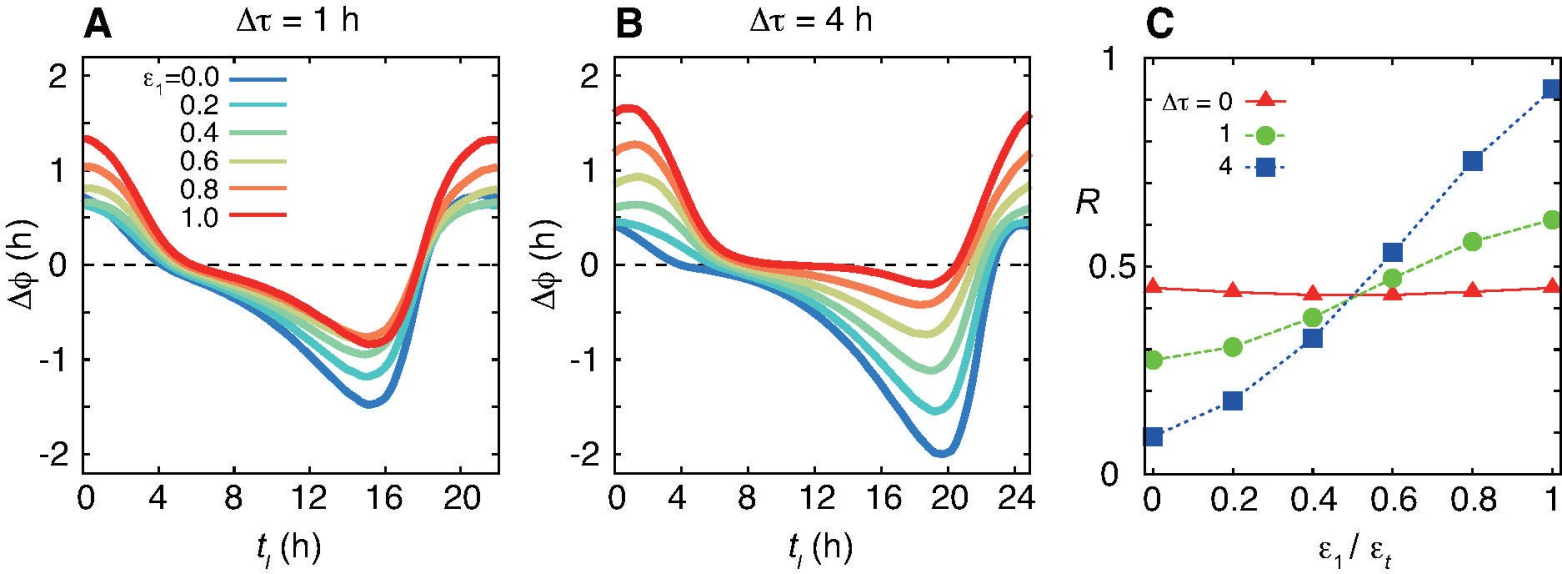
Diverse PRC shapes resulting from different ratios of light-induced transcription rates. (A), (B) PRCs for light signals with (A) Δ*τ* = 1 h and (B) Δ*τ* = 4 h. Different lines indicate results with the different ratios of light-induced transcription rates *ϵ*_1_/*ϵ*_*t*_ where ϵ_*t*_ = ϵ_1_ + ϵ_2_. (C) Area fraction of advance zone *R* in Eq (3) as a function of *ϵ*_1_/*ϵ*_*t*_ for different values of Δ*τ*. In (A)-(C), the total light-induced transcription rate is fixed as ϵ_*t*_ = ϵ_1_ + ϵ_2_ = 1. The light-induced transcription rate of *P2* is, then, ϵ_2_ = ϵ_*t*_ − ϵ_1_. We set ${\bar{\tau }}_{1}=1$ h and ${\bar{\tau }}_{2}=1.5$ h in Eq (1a). The values of the other parameters are described in S1 Text.

If the peak time difference between the two NFLs is small, PRC shapes by different ratios *ϵ*_1_/*ϵ*_*t*_ are almost the same (Fig 3A and 3C). In contrast, if the peak time difference is large, the PRC shape strongly depends on *ϵ*_1_/*ϵ*_*t*_ (Fig 3B and 3C). When *ϵ*_1_/*ϵ*_*t*_ is small, the PRC includes a large delay zone, i.e. a small advance zone, resulting in small *R*. As *ϵ*_1_/*ϵ*_*t*_ increases, the area of advance zone gradually expands and *R* reaches close to one. As mentioned in the introduction, the magnitude of light-induced transcription of *Per1* mRNA in rat and hamster SCNs has been found to be greater than that of *Per2* mRNA [12,26]. Hence, PRCs with a larger advance zone than delay zone in these animals [4,51] coincide with the prediction of the simulation. In summary, our theoretical results indicate that different PRC shapes can be generated depending on the ratio of light-induced transcription rates between two repressors in dual NFLs in the presence of time difference in their expression peaks.

### Entrainment to a 24-hour LD cycle

**Fig 4:**
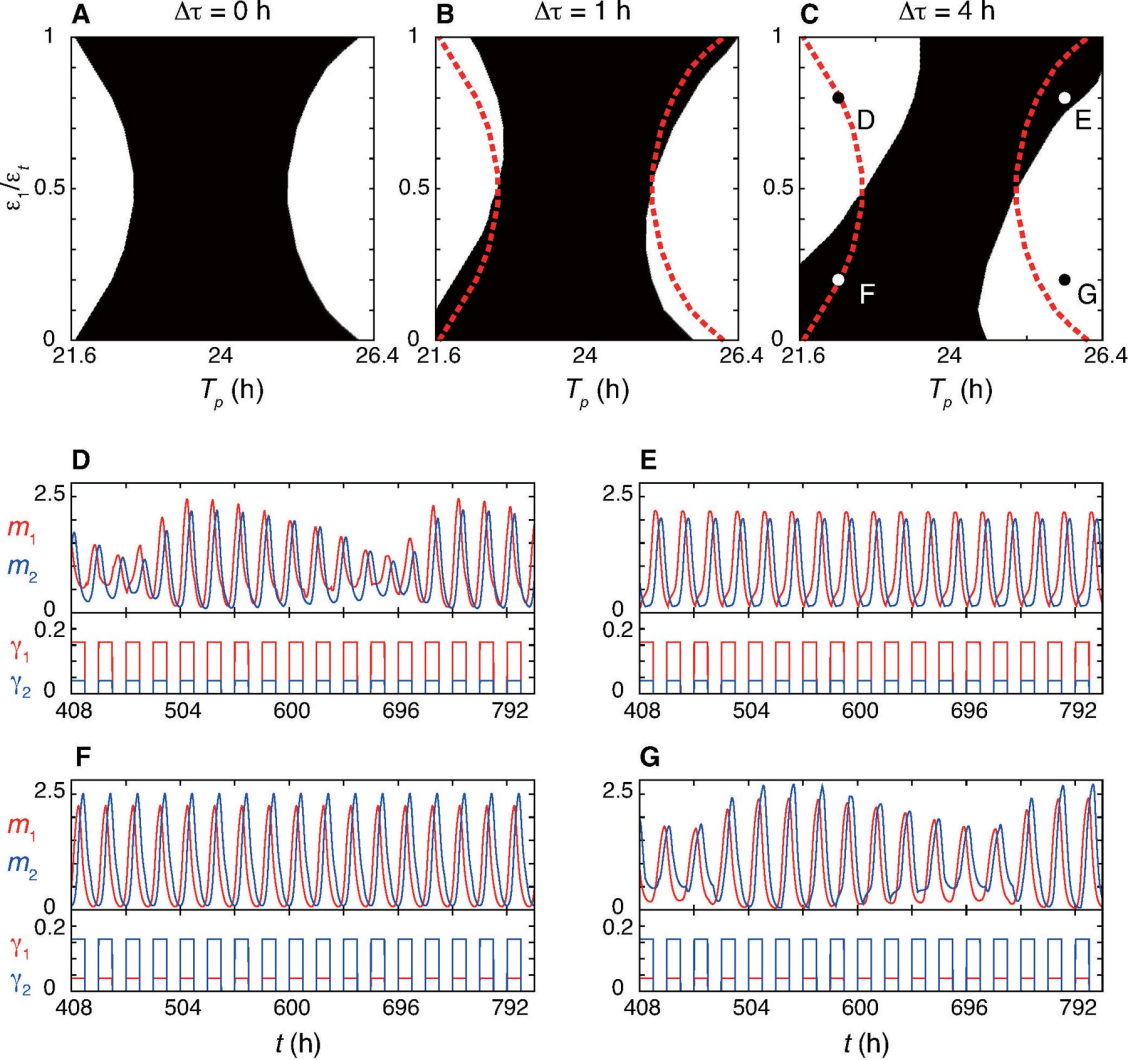
Entrainment to a 24-hour light-dark (LD) cycle determined by the ratio of light-induced transcription rates between two NFLs. (A)-(C) Dependence of the range of entrainment (black) on the peak time difference: (A) Δ*τ* = 0 h, (B) 1 h and (C) 4 h. The horizontal axis is the autonomous period *T*_*p*_. The vertical axis is the ratio of light-induced transcription rate *ϵ*_1_/*ϵ*_*t*_. In the white region, stable phase lock does not occur as shown in (D) and (G). Red dotted lines in (B) and (C) mark the boundaries of range of entrainment for Δ*τ* = 0 as shown in (A). The total rate is ϵ_*t*_ = ϵ_1_ + ϵ_2_ = 0.2. Then, ϵ_2_ = 0.2 − ϵ_1_. *T*_*p*_ is changed by varying the time delay parameters in translation, see S1 Text. (D)-(G) Time series of (top) *m*_1_ and *m*_2_, and (bottom) *γ*_1_ and *γ*_2_. The values of *ϵ*_1_/*ϵ*_*t*_ and *T*_*p*_ are indicated in (C): (D) *ϵ*_1_/*ϵ*_*t*_ = 0.8, *T*_*p*_ = 22.2, (E) *ϵ*_1_/*ϵ*_*t*_ = 0.8, *T*_*p*_ = 25.8, (F) *ϵ*_1_/*ϵ*_*t*_ = 0.2, *T*_*p*_ = 22.2 and (G) *ϵ*_1_/*ϵ*_*t*_ = 0.2, *T*_*p*_ = 25.8. Other parameter values are listed in S1 Text.

As described in the previous section, the ratio of light-induced transcription rates between the two NFLs in the presence of the time difference between expression peaks of the two repressors Δ*τ* controls the magnitude and direction of phase shifts at each subjective time. The magnitude of phase shifts by light signals determines whether a circadian clock with a certain autonomous period can entrain to an LD cycle. Hence, we identify the ratio of light-induced transcription rates that enables a circadian clock with the peak time difference Δ*τ* and autonomous period *T*_*p*_ to entrain to a 12:12 LD cycle (Fig 4). We control *T*_*p*_ in this analysis by changing the time delays in translation *T*_1_ and *T*_2_ in Eq (1b) (S1 Text). As in the previous section, we fix the total rate as ϵ_*t*_ = ϵ_1_ + ϵ_2_, and change the value of ϵ_1_ to examine entrainment.

Fig 4A–4C shows the range of entrainment in the *T*_*p*_ and *ϵ*_1_/*ϵ*_*t*_ parameter space. For the identical NFLs Δ*τ* = 0, the range of entrainment is symmetric about *ϵ*_1_/*ϵ*_*t*_ = 0.5 (Fig 4A). To maximize the range of entrainment along the *T*_*p*_ axis, the ratio of *P1* induction should be either close to one or close to zero. This suggests that if the period difference is large, only one NFL should be light-responsive to attain entrainment with a fixed total rate ϵ_*t*_.

With the peak time difference Δ*τ* > 0, the range of entrainment becomes asymmetric about *ϵ*_1_/*ϵ*_*t*_ = 0.5 (Fig 4B and 4C). If *T*_*p*_ is shorter than the period of the LD cycle, the value of *ϵ*_1_/*ϵ*_*t*_ needs to be small for entrainment to occur (Fig 4B and 4C). In other words, stronger light induction of *P2* than *P1* is necessary to delay the clock (Fig 4D and 4F). Conversely, if *T*_*p*_ is longer than the period of the LD cycle, the value of *ϵ*_1_/*ϵ*_*t*_ needs to be greater than 0.5 for such slower clocks to entrain to the LD cycle by increasing their speed (Fig 4B–4G). Thus, the repressor that should be induced by light signals more strongly than the other is determined based on the complementary contributions of the two NFLs and the autonomous period. In addition, the range of entrainment with peak time difference Δ*τ* > 0 partly encompasses that with Δ*τ* = 0, as shown in the bottom left (*T*_*p*_ < 24 and *ϵ*_1_/*ϵ*_*t*_ < 0.5) and top right (*T*_*p*_ > 24 and *ϵ*_1_/*ϵ*_*t*_ > 0.5) corners of Fig 4B and 4C. Thus, a light-induced transcription rate of *P1* mRNA satisfying *ϵ*_1_/*ϵ*_*t*_ > 0.5 (*ϵ*_1_/*ϵ*_*t*_ < 0.5) in the presence of peak time difference can entrain a circadian clock with a longer (shorter) autonomous period compared to its absence. The ratio *ϵ*_1_/*ϵ*_*t*_ also determines the peak times of *P1* and *P2* mRNAs in an entrained rhythm (S9 Fig). Taking a human autonomous period (~ 24.5 hours [52]) as an example, *P1* and *P2* mRNAs peak earlier as the ratio *ϵ*_1_/*ϵ*_*t*_ increases (S9A–S9D Fig). Since the peak time of *P1* and *P2* mRNAs in a LD cycle would represent human chronotypes [53], this result suggests that the variations in the ratio of light-induced transcription rates between the two *Per* genes can be one of the determining factors of human chronotypes.

Taken together, two interacting NFLs have complementary effects on phase shifts and, as a result, the ratio of their light-induced transcription rates determines entrainability to LD cycles.

## Discussion

In this study, we examined two interacting NFLs for genetic oscillations to better understand the mammalian circadian clock system. In mammals, *Per1* and *Per2* consist of interacting NFLs that are responsible for circadian genetic oscillations. Peak time differences between *Per1* and *Per2* expression have been observed in the central pacemaker tissue, SCN [12–16]. Furthermore, both *Per1* and *Per2* play key roles in entrainment to LD cycles [7,24,26,54]. The lack of one of the two *Per* genes resulted in irregularity in autonomous rhythms in SCN, and increased sensitivity of circadian rhythms to genetic background [10,11], suggesting that the dual NFLs of *Per* genes enhance robustness of the circadian clock. However, besides their role as redundant transcriptional repressors, functional differences between *Per1* and *Per2* gene products have yet to be revealed. Our results predict the novel and distinct roles of *Per1* and *Per2* in phase responses to light signals: light induction of *Per1* mRNA mainly contributes to phase advance, whereas induction of *Per2* mRNA contributes to phase delay.

Our analysis revealed that difference in passage time at which the levels of PER1 and PER2 proteins become higher or lower than the dissociation constant to E-box is key to their functional differentiation in phase responses to light signals. A previous immunohistochemical study observed that the PER1 and PER2 protein expression peaked at CT12 in the mouse SCN [33]. Since the modification such as phosphorylation of PER proteins influences their repressor assembly and activities [49,55], the complementarity of PER1 and PER2 proteins must be reflected by the kinetics of their presence on E-box. In fact, a Chip-seq analysis using mouse liver indicated that PER2 protein stays at the E-boxes of circadian clock gene promoters 1~4-hour later than PER1 [56], suggesting that the timepoint of PER2 protein levels below the dissociation constant is later than that of PER1. Similar experiments using the SCN would be worth to evaluate our conclusion.

We addressed phase responses of circadian rhythms to light signals in a single SCN neuron, since its phase responses is the basis of those at tissue and individual levels. In the SCN, neurons interact with each other via various neurotransmitters, such as vasoactive intestinal peptide and arginine vasopressin [57]. In addition to keeping precise ticking [10], the intercellular coupling may also modulate responsiveness of oscillators to environmental signals [58,59]. Phase responses of coupled circadian oscillators have been quantified by neuronal firing rate in SCN slices [21,60]. Induction of *Per1* mRNA in the rat SCN slices by glutamate advanced the circadian phase of the neuronal firing rate [21]. Importantly, the phase advance by glutamate was inhibited in the presence of an antisense oligodeoxynucleotide against *Per1* mRNA, but not by antisense oligodeoxynucleotide against *Per2* [21]. Thus, these results are consistence with our theoretical prediction in a single neuron.

The complementary contributions of interacting NFLs to phase responses may underlie the diverse PRC shapes observed in different animal species. Even within mammals, the area ratios of advance to delay zones in PRCs differ among species [4]. One way to modify the shape of a PRC is by tuning reaction parameters within NFLs, such as transcription and degradation rates of *Per1* and *Per2* mRNAs [34], although such changes may also affect the period and amplitude of oscillation. Another possibility could be to gate light input signals to the two *Per* genes in the SCN depending on time of day, to modulate the ratio of advance and delay zones [61,62]. However, such gating would require an elaborate clock-dependent mechanism. On the other hand, our current study revealed a new mechanism to create diverse PRC shapes only by changing the ratio of light-induced transcription rates between *Per1* and *Per2* in the presence of peak time difference in the SCN.

The PRC shape has been considered important for stable entrainment to LD cycles [4,7,63]. With a phase oscillator model, a previous theoretical study analyzed an optimal PRC shape for stable entrainment to LD cycles, when a circadian clock system includes two light-sensitive reactions [64]. Interestingly, if there is a phase difference between two variables influenced by these light-sensitive reactions, to maximize entrainability, each of these should contribute to phase responses in a complementary way, with one variable mainly contributing to phase advance, whereas the other contributes to phase delay [64], like *P1* and *P2* in the current study. However, the abstract phase oscillator model cannot address questions whether and how such PRCs are realized by gene regulations in the circadian clock. By modeling gene regulatory network, our current study offers a possible mechanism for complementary phase responses with the two light-responding clock genes, *Per1* and *Per2*. Mammalian circadian clocks also include other redundant NFLs such as *Cry1* and *Cry2*, and *Rev-erbα* and *Rev-erbβ* [40]. A future modeling approach similar to the current one might reveal functional differentiation of these redundant feedback loops. In addition, we notice that dual NFLs with a 4-hour difference between expression peaks of the two repressors generate an interval in a PRC where phase shifts are close to zero, termed a dead zone [4,34], but those with a 1-hour difference do not (Fig 3A and 3B). Thus, the dual NFLs of *Per1* and *Per2* would be responsible for not only diverse PRC shapes but also characteristics of type-1 PRC observed in mammals.

In rats and hamsters, the area of the advance zone in a PRC for behavioral rhythms is larger than that of the delay zone [4,51]. The current theoretical results predict that such larger advance zones result from the greater light-induced transcription of *Per1* than *Per2*. Consistent with this prediction, *Per1* expression in their SCNs was found to be strongly induced by short light signals at subjective night, whereas *Per2* expression was weaker than *Per1* [12,26]. Furthermore, our model predicts that if the period of an LD cycle is shorter than the autonomous period of animals, stronger induction of *Per1* than *Per2* mRNAs is required to entrain to the LD cycle, and vice versa. Experimental results for rats and hamsters are consistent with this prediction (S1 Table) [26,54], suggesting that the ratio of light-induced transcription rates between *Per1* and *Per2* would be constrained by the difference between the autonomous and the LD periods in these animals.

In mice, administration of light signals that caused phase delays in behavioral rhythms at subjective night resulted in weak induction of *Per1* mRNA but strong induction of *Per2* mRNA in the dorsal region of the SCN (S1 Table) [23,25]. The current model suggests that such strong *Per2* induction in the dorsal SCN may underlie the larger delay zone in mouse PRCs [4,7]. Conversely, when a light signal caused phase advance in behavioral rhythms at subjective late night, the induction levels of *Per1* mRNA were higher than those of *Per2* mRNA in the dorsal region [23,25]. Thus, the correlations between relative induction levels of *Per* genes in the dorsal region of the mouse SCN and the direction of phase shifts are consistent with current theory. In the ventral region of the mouse SCN, however, *Per1* mRNA was induced by light signals that caused a phase advance [23], phase delays [19,65] and even no phase shift [23] in behavioral rhythms (S1 Table), probably reflecting a major role of this region in the reception of light signals from retina. In summary, with few exceptions, experimental observations from mammals have consistently indicated that the induction of *Per1* mRNA contributes to phase advance, whereas that of *Per2* mRNA contributes to phase delay.

Although autonomous rhythms in SCN cells are compromised in *Per1* or *Per2* deficient mice, their behavioral rhythms are almost indistinguishable from WT [10,11]. A previous experiment measured the PRCs of mice lacking either functional *Per1* or *Per2* [7]. These mutant mice possessed only a single NFL, but this remaining NFL could cause both phase advance and delay, according to our theoretical results. Therefore, an experiment to verify the current model predictions would require the inhibition of light inputs into one of the two *Per* genes without any effect on the circadian expression of both genes. Light signals lead to the activation of cAMP response element binding protein (CREB), which induces transcription of *Per1* and *Per2* after binding to the CRE element in their promoter regions. Since circadian expression of the two *Per* genes depends not on CRE but on the E-box element in their own promoters, isolation of CRE mutant mice of *Per1* or *Per2* should satisfy the above requirement. Alternatively, the complementary contributions of *Per1* and *Per2* to phase responses could be examined by specific induction of one of the two *Per* genes. Although such chemical inducers are not available at present, the current study should provide motivation for their screening for the treatment of circadian rhythm disorders and adjustment for different chronotypes. Previous genome-wide association studies associated morningness with single nucleotide polymorphisms near human *PER1* and *PER2* loci [66,67], confirming the relevance of these two *PER* genes to human chronotypes. The current study predicts that a chemical compound that specifically induces *PER1* mRNA may be able to cause phase advances only, regardless of administration time, and could be useful for the treatment of delayed sleep phase syndrome [68,69]. Conversely, a compound that targets *PER2* specific induction might be used to delay the clock at any zeitgeber time, which would be useful for the treatment of advanced sleep phase syndrome [69,70]. Thus, as our simulations suggested, the administration of such compounds may adjust the phase of entrainment to be more desirable for daily life.

Although we focused on the interlocked NFLs of the two *Per* genes, the mammalian circadian clocks include other positive and negative feedback loops [40,71]. Importantly, the expression of clock genes containing E-box in their promoters, such as *Per1* and *Rev-erb*s, tends to peak at earlier time of a day, whereas the expression of those containing ROR element in their promoters, such as *Bmal1*, peaks at later time [16,40,72]. The different phases of these clock gene expression would reflect their specific functions important for sustaining robust circadian oscillation [31,48,50] and entrainment. How these multiple feedback loops modulate the complementary phase responses by the two *Per* genes is an important question that should be addressed in future study. Since functional differentiation between PER1 and PER2 proteins is predicted to depend on the similar repression activities of both proteins, it is not necessary to revise our conclusion as long as the other feedback loops do not affect the relative repression activities.

In conclusion, we revealed the complementary contributions of two interacting NFLs to phase responses and entrainment. Our results suggest that the peak time differences between transcription factors in the multiple feedback loops lead to their functional differentiation. Such functional differentiation may be key to understanding temporal dynamics in complex gene regulatory networks.

## Supporting information

S1 Table Correlation between the light-induced transcription of *Per1* and *Per2* mRNAs in the SCN, and phase responses of behavioral rhythms to light signals in previous experiments for (A) rats and hamsters, and (B) mice.
(PDF)

S1 Text Method details. (PDF)

S1 Fig Relations between the difference in transcriptional time delays Δ*τ*, peak time difference, and phase of first Fourier modes. (A) Time series of *P1* (*m*_1_: red dotted) and *P2* (*m*_2_: blue dotted) mRNAs. The solid red and blue lines indicate the first Fourier modes of *m*_1_ and *m*_2_, respectively. Δ*t*_*p*_ is the peak time difference between *m*_1_ and *m*_2_. Δ*φ* is the phase difference between the first Fourier modes. (B) Correlation between Δ*τ* and Δ*t*_*p*_. (C) Correlation between Δ*τ* and Δ*φ*. In (B) and (C), dotted diagonal lines indicate *y* = *x*.
(TIFF)

S2 Fig Dependence of the amplitude and period of oscillation on time delays in translation *T*_*i*_. (A) (Top) Timeseries of *p*_1_ (red solid), *p*_2_ (blue solid), and *p*_1_^*n*^ + *p*_2_^*n*^ (green solid). The black horizontal line indicates the dissociation constant *K*. Green horizontal line indicates *K*^*n*^. Vertical lines indicate the time at which *p*_1_^*n*^ + *p*_2_^*n*^ becomes larger (red) or smaller (blue) than *K*^*n*^. Log scale in left y-axis for *p*_1_^*n*^ + *p*_2_^*n*^ and linear scale in right y-axis for *p*_1_ and *p*_2_. (Bottom) Green marks × and + indicate time at which *p*_1_^*n*^ + *p*_2_^*n*^ becomes smaller and larger than *K*^*n*^, respectively. Red open circles indicate time at which *p*_1_ becomes larger than *K*. Blue open triangles indicate time at which *p*_2_ becomes smaller than *K*. (B) Timeseries of the light-independent transcription rate of *P1* mRNA in Eq (1a) (black dotted), *p*_1_ (red solid) and *p*_2_ (blue solid). The solid horizontal line indicates the dissociation constant *K*. The dotted horizontal line indicates *β*/2 where *β* = 1. Red and blue circles indicate passage time of *p*_1_ and *p*_2_ for *K*, respectively. Red triangles and blue squares indicate the times at which light-independent transcription rate decreases to and increases to *β*/2, respectively. These passage times are plotted in (C). (C) Passage time of light-independent transcription rate of *P1* mRNA in Eq (1a) for *β*/2 as a function of the passage time of *p*_1_ or *p*_2_ for *K*. Blue squares indicate the relation between the time at which the transcription rate increases to *β*/2 and the one at which *p*_2_ becomes smaller than *K*. Red triangles indicate the relation between the time at which the transcription rate decreases to *β*/2 and the one at which *p*_1_ becomes larger than *K*. The dotted line indicates y = x + τ_1_. (D), (E), (H), (I) Timeseries of (top) mRNAs *m*_*i*_ and proteins *p*_*i*_, and (bottom) light-independent transcription rate for the different values of time delays in translation *T*_*i*_. (D) *T*_1_ = 3 h and (E) *T*_1_ = 7 h with *T*_2_ = 5 h. (H) *T*_2_ = 3 h and (I) *T*_2_ = 7 h with *T*_1_ = 5 h. In the bottom panels, timeseries of *p*_1_ and *p*_2_ are also plotted. Solid horizontal lines indicate *K*. Dotted horizontal lines indicate *β*/2. (F), (J) Dependence of the duration where the light-independent transcription rate of *P1* mRNA is larger than *β*/2 on (F) *T*_1_ and on (J) *T*_2_. (G), (K) Dependence of the duration where the light-independent transcription rate of *P1* mRNA is smaller than *β*/2 on (G) *T*_1_ and on (K) *T*_2_. Insets in (F) and (G) show the definitions of these durations. (L), (M) Dependence of *p*_2_ amplitude on (L) *T*_1_ and (M) *T*_2_. The inset in (L) shows the definition of *p*_2_ amplitude. (N), (O) Dependence of the time interval of *p*_2_ increase (red open circles) and the period of oscillation (blue filled circles) on (N) *T*_1_ and (O) *T*_2_. The inset in (N) shows the definition of the time interval of *p*_2_ increase (red arrow) and the period of oscillation (blue arrow).
(TIFF)

S3 Fig Changes in light-independent transcription rate of *P1* mRNA by light induction of *P1* or *P2* mRNA. (A)-(D) Timeseries of light-independent transcription rate of *P1* mRNA in Eq (1a) (black), levels of P1 (red) and P2 (blue) proteins in the presence of a light signal. Each panel corresponds to Fig 2G–2J in the main text. A light signal is administered at (A), (B) *t*_*l*_ = 2 h, (C) *t*_*l*_ = 11 h and (D) *t*_*l*_ = 16 h. In (A) and (C), only *P1* mRNA is induced by the light signal. In (B) and (D), only *P2* mRNA is induced by the light signal. Gray lines indicate timeseries in the absence of a light signal. Solid horizontal lines indicate the dissociation constant *K*. Dotted horizontal lines indicate *β*/2 with *β* = 1.
(TIFF)

S4 Fig Dependence of complementary phase responses on time delays in translation. (A)-(C) Time series of mRNAs and proteins for (A) *T*_2_ = 1 h, (B) *T*_2_ = 2.2 h and (C) *T*_2_ = 3.4 h. Horizontal lines indicate the dissociation constant *K* in Eq (1a). For better comparison, time is normalized by the autonomous period *T*_*p*_. (D) Dependence of the area fraction *R* (red and blue dotted) and index *c* (green solid) on the ratio of total time delays in each negative feedback loop. *T*_2_ changes from 1 to 7 hours with all the other parameters fixed. In all panels, *τ*_1_ = 1 h, *τ*_2_ = 5 h and *T*_1_ = 5 h.
(TIFF)

S5 FigDependence of complementary phase responses on parameters in light-induced transcription.(A)-(I) Dependence of PRCs on (A)-(C) time delays ${\bar{\tau }}_{i}$, (D)-(F) light induced transcription rate ϵ and (G)-(I) light duration *T*_*d*_ in Eqs (1a) and (2). The red lines indicate PRCs with ϵ_1_ = ϵ and *ϵ*_2_ = 0. The blue lines indicate PRCs with ϵ_1_ = 0 and *ϵ*_2_ = *ϵ*. In (A)-(C), ϵ = 0.5 and *T*_*d*_ = 1 h. In (D)-(F), ${\bar{\tau }}_{1}=1$ h, ${\bar{\tau }}_{2}=5$ h and *T*_*d*_ = 1 h. In (G)-(H), $\epsilon =0.5,\;{\bar{\tau }}_{1}=1$ h, and ${\bar{\tau }}_{2}=5$ h. (J), (K) Time series of mRNAs and proteins in the presence of a light signal (red and blue lines). Light induction of only (J) *P1* or (K) *P2* mRNA. Horizontal lines indicate the dissociation constant *K* in Eq (1a). Gray lines indicate time series in the absence of a light signal. *T*_*d*_ = 1 h.(TIFF)

S6 Fig Dependence of complementary phase responses on reaction parameters. (A)-(E) Dependence of the area fraction *R* and complementarity index *c* on ratios of nondimensional reaction parameters. Results of the two NFLs in an extended model Eq. (S2) in S1 Text. Dependence on (A) the ratio of light-independent transcription rates *b* = *β*_2_/*β*_1_, (B) dissociation constants *κ*_21_/*κ*_12_ = *K*_2_/*K*_1_, (C) degradation rates of mRNAs *a* = *α*_2_/*α*_1_, (D) translation rates *f*_2_/*f*_1_ = *ν*_2_/*ν*_1_, and (E) degradation rates of proteins *h*_2_/*h*_1_ = *μ*_2_/*μ*_1_. In (B), we fix *κ*_12_ =1 and change *κ*_21_ in Eq. (S2). In (D), *f*_1_ = 3.47 and we change *f*_2_. In (E), *h*_1_ = 1 and we change *h*_2_. (F) Timeseries of mRNAs and proteins with (top) *b* = *β*_2_/*β*_1_ = 0.5 and (bottom) *b* = *β*_2_/*β*_1_ = 1 in the absence of light signals. Dotted horizontal lines indicate the value of the dissociation constant *κ* = *κ*_*ij*_ (*i*, *j* = 1, 2). (G), (H) Dependence of *R* and *c* on the Hill coefficients (G) *n*_1_ and (H) *n*_2_ in Eq. (S2). (I)-(K) Dependence of *R* and *c* on the nondimensional parameter *f* = *νβ*/(*α*^2^*K*) for different values of *h* = *μ*/*α* in Eq. (S3) in S1 Text. (I) *μ*/*α* = 0.5, (J) *μ*/*α* = 1, and (K) *μ*/*α* = 2. Gray shades indicate the parameter regions where a steady state is stable and there is no limit cycle solution. Green shades indicate the parameter regions where the shape of PRCs is discontinuous type-0 (inset in (I)). See S1 Text for derivation of a nondimensional form of Eq (1) and values of parameters.
(TIFF)

S7 Fig Period, amplitude and phase responses of a single negative feedback loop with multiple states of mRNA and protein. (A) Schematic of a single negative feedback loop including a linear chain of mRNA and protein state transitions. *η* is the time constant of mRNA state transition and *λ* is that of protein state transition. The functional protein *p*_1*r*_ represses its own transcription. (B) Time series of the functional mRNA *m*_1*u*_ available for translation for different values of *η*. The value of *η* changes from 0.3 (blue) to 0.7 (red) with the step size of 0.05. (C) Dependence of period and amplitude of oscillation on *η*. The amplitude of functional protein *p*_1*r*_ is plotted. (D) Phase response curves (PRCs) for the light induction of *m*_11_ with two different values of *η*. (E) Time series of the functional mRNA *m*_1*u*_ for different state number *u*. The value of *u* changes from 2 (red) to 7 (blue). (F) Dependence of period (red circles) and amplitude (blue squares) of oscillation on *u*. (G) PRCs for the light induction of *m*_11_ with different values of *u*. Dotted horizontal lines in (C) and (F) indicate period of 24 hours as a reference. In (D) and (G), phase shift Δ*ϕ* and administration time *t*_*l*_ are indicated in the unit of circadian time (CT) for better comparison. The values of other parameters are listed in S1 Text.
(TIFF)

S8 Fig Complementary phase responses in dual negative feedback loops including multiple states of mRNA and protein. (A) Schematic of dual negative feedback loops including linear chains of mRNA and protein state transitions. *η*_1_ is the time constant of *P1* mRNA state transition and *η*_2_ is that of *P2* mRNA state transition. *λ* is the time constant of protein state transition. Functional proteins *p*_1*r*_ and *p*_2*r*_ repress their own and opponent’s transcription. (B) Dependence of peak time difference between *m*_1*u*_ and *m*_2*w*_ on the ratio of time constants of state transition *η*_2_/*η*_1_. Results for different values of state number of *P2* mRNA *w* are shown. Dotted horizontal line indicates 4-hour peak time difference as a reference. (C) Dependence of the amplitude of *p*_2*r*_ on *η*_2_/*η*_1_ for different values of *w*. (D), (E) Time series of functional mRNAs and proteins for (D) *w* = 4 and *η*_2_/*η*_1_ = 0.583, and (E) *w* = 5 and *η*_2_/*η*_1_ = 0.833. Dotted horizontal lines indicate the dissociation constant *K*. (F), (G) Phase response curves to light signals for (F) *w* = 4 and *η*_2_/*η*_1_ = 0.583, and (G) *w* = 5 and *η*_2_/*η*_1_ = 0.833. In (B)-(G), *u* = 3. The values of other parameters are listed in S1 Text.
(TIFF)

S9 Fig Effect of light-induced transcription rates of *P1* and *P2* mRNAs on the phase of entrainment. (A)-(C) Time series of *P1* (red) and *P2* (blue) mRNAs in the presence of a 12:12 light-dark (LD) cycle. The ratio of light-induced transcription rates is (A) ϵ_1_/ϵ_*t*_ = 0.95, (B) ϵ_1_/ϵ_*t*_ = 0.5 and (C) ϵ_1_/ϵ_*t*_ = 0.2. Entrained rhythms of *m*_1_ and *m*_2_ are plotted. (D) Dependence of peak time of *P1* and *P2* mRNAs on ϵ_1_/ϵ_*t*_. The gray shade region indicates time interval where light signal is off. The autonomous period is 24.51 hours with *T*_1_ = *T*_2_ = 4.82 h in Eq (1b).
(TIFF)

S1 File Simulation codes. (ZIP)
