# Supplementary material for: Complementary phase responses via functional differentiation of dual negative feedback loops
Source: PLoS Comput Biol. 2021 Mar 8;17(3):e1008774. doi: 10.1371/journal.pcbi.1008774 (PMC7971863; doi:10.1371/journal.pcbi.1008774)
Supplement: S1 Table — Correlation between the light-induced transcription of Per1 and Per2 mRNAs in the SCN, and phase responses of behavioral rhythms to light signals in previous experiments for (A) rats and hamsters, and (B) mice. (PDF) [file pcbi.1008774.s001.pdf]

S1 Table Correlation between the light-induced transcription of *Per1* and *Per2* mRNAs in the SCN, and phase responses of behavioral rhythms to light signals in previous experiments for (A) rats and hamsters, and (B) mice.

A

	free-running period	T cycle	induction in entrained SCN	refs.**
rat	24.4~24.9 h	12:12	*Per1*	51, 54
		12.5:12.5	*Per2*	
hamster	24.04 h	1-h light pulse every 23.33 h	*Per1*	4, 26
		1-h light pulse every 24.67 h	increased *Per2* mRNA stability*	

* Stable *Per2* mRNA could prolong protein production similar to the induction of mRNA, leading to elevation of PER2 protein levels.

** Reference number in the main text.

B

mouse	induction				
	light signal	dorsal SCN	ventral SCN	phase responses in behavioral rhythms	refs.*
	CT 22	*Per1*	*Per1*	advance	23
	CT 14	*Per2*	*Per1* & *Per2*	delay	

* Reference number in the main text.
