# Supplementary material for: Complementary phase responses via functional differentiation of dual negative feedback loops
Source: PLoS Comput Biol. 2021 Mar 8;17(3):e1008774. doi: 10.1371/journal.pcbi.1008774 (PMC7971863; doi:10.1371/journal.pcbi.1008774)
Supplement: S1 Text — (PDF) [file pcbi.1008774.s002.pdf]

Complementary phase responses via functional differentiation of dual negative feedback loops

Koichiro Uriu, Hajime Tei

Graduate school of Natural Science and Technology, Kanazawa University, Kakuma-machi, Kanazawa, 920-1192, Japan

S1 Text

Derivation of nondimensional equations

In the main text, we adopt the model Eq. (1) where the values of reaction parameters in the two NFLs are the same. Molecular characteristics of *Per1* and *Per2* gene products, such as translation rate and degradation rate, are considered similar due to the similarity in their protein sequences. To examine whether the similarity in reaction parameter values between *Per1* and *Per2* is relevant to their complementary contributions to the phase responses, here we consider an extended model including differences of the values of these reaction parameters in the two NFLs. We analyze the dependences of phase responses on the ratio of reaction parameters between the two NFLs. To clarify how these ratios are involved in the delay differential equations, we derive their nondimensional form below.

We first incorporate the differences in reaction parameters between the two NFLs into equations:

$$\frac{dm_i(t)}{dt} = \frac{\beta_i}{1 + (p_1(t-\tau_i)/K_{i1})^{n_i} + (p_2(t-\tau_i)/K_{i2})^{n_i}} + \gamma_i(t-\bar{\tau}_i) - \alpha_i m_i(t), \qquad (S1a)$$

$$\frac{dp_i(t)}{dt} = \nu_i \cdot m_i(t-T_i) - \mu_i \cdot p_i(t), \qquad (S1b)$$

where $i \in \{1,2\}$. We then introduce the nondimensional variables M_i, P_i and $\tilde{t}$ as: $m_1 = (\beta_1/\alpha_1)M_1,\ m_2 = (\beta_1/\alpha_1)M_2,\ p_1 = K_{11}P_1,\ p_2 = K_{11}P_2,\ t = \tilde{t}/\alpha_1$. Substituting these nondimensional variables into Eq. (S1), we obtain:

$$\frac{dM_1(\tilde{t})}{d\tilde{t}} = \frac{1}{1 + P_1(\tilde{t}-\tilde{\tau}_1)^{n_1} + (P_2(\tilde{t}-\tilde{\tau}_1)/\kappa_{12})^{n_1}} + g_1(\tilde{t}-\bar{\bar{\tau}}_1) - M_1(\tilde{t}), \qquad (S2a)$$

$$\frac{dM_2(\tilde{t})}{d\tilde{t}} = b\frac{1}{1 + (P_1(\tilde{t}-\tilde{\tau}_2)/\kappa_{21})^{n_2} + \left(P_2(\tilde{t}-\tilde{\tau}_2)\right)^{n_2}} + g_2(\tilde{t}-\bar{\bar{\tau}}_2) - aM_2(\tilde{t}), \qquad (S2b)$$

$$\frac{dP_1(\tilde{t})}{d\tilde{t}} = f_1 \cdot M_1\left(\tilde{t}-\tilde{T}_1\right) - h_1 \cdot P_1(\tilde{t}), \qquad (S2c)$$

$$\frac{dP_2(\tilde{t})}{d\tilde{t}} = f_2 \cdot M_2\left(\tilde{t}-\tilde{T}_2\right) - h_2 \cdot P_2(\tilde{t}), \qquad (S2d)$$

where we introduce the following nondimensional parameters:

$\kappa_{12} = K_{12}/K_{11},\ \kappa_{21} = K_{21}/K_{11},\ \kappa_{22} = K_{22}/K_{11},$

$b = \beta_2/\beta_1,\ a = \alpha_2/\alpha_1,\ f_1 = \nu_1\beta_1/(\alpha_1{}^2K_{11}),\ f_2 = \nu_2\beta_1/(\alpha_1{}^2K_{11}),$

$h_1 = \mu_1/\alpha_1\ \ h_2 = \mu_2/\alpha_1,\ g_1(t) = \gamma_1(t)/\beta_1,\ g_2(t) = \gamma_2(t)/\beta_1,$

$\tilde{\tau}_i = \alpha_1\tau_i,\ \tilde{T}_i = \alpha_1 T_i,$ and $\bar{\bar{\tau}}_i = \alpha_1\bar{\tau}_i$.

In addition, we assume $\kappa_{22} = K_{22}/K_{11} = 1$ in Eq. (S2) to simplify the analysis.

From Eq. (S2), we obtain the ratios of reaction parameters: ratio of light-independent

transcription rates b, degradation rates of mRNAs a, translation rates $f_2/f_1 = \nu_2/\nu_1$, and degradation rates of proteins $h_2/h_1 = \mu_2/\mu_1$.

If we assume $\beta_1 = \beta_2$, $\alpha_1 = \alpha_2$, $\nu_1 = \nu_2$, $\mu_1 = \mu_2$, $K_{11} = K_{12} = K_{21} = K_{22}$ and $n_1 = n_2$ in Eq. (S1), Eq. (S2) becomes:

$$\frac{dM_i(\tilde{t})}{d\tilde{t}} = \frac{1}{1 + P_1(\tilde{t} - \tilde{\tau}_i)^n + P_2(\tilde{t} - \tilde{\tau}_i)^n} + g_i(\tilde{t} - \bar{\bar{\tau}}_i) - M_i(\tilde{t}), \qquad (S3a)$$

$$\frac{dP_i(\tilde{t})}{d\tilde{t}} = f \cdot M_i(\tilde{t} - \tilde{T}_i) - h \cdot P_i(\tilde{t}). \qquad (S3b)$$

Eq. (S3) is a nondimensional form of Eq. (1) in the main text. We use Eqs. (S2) and (S3) to analyze parameter dependence of PRCs in S6 Fig.

Entrainment analysis

We determine the ratio of light-induced transcription rates between *P1* and *P2* that enables the circadian clock with the certain values of peak time difference $\Delta\tau = \tau_2 - \tau_1$ and autonomous period T_p to entrain to the 24-hour LD cycle in Fig. 4. To control T_p with a given peak time difference $\Delta\tau$, we vary the delays in translation T_1 and T_2 simultaneously in Eq. (1). Specifically, in Fig. 4A-C, we set $\tau_1 = \tau_2 = 2.9$ when $\Delta\tau = 0$ (Fig. 4A), $\tau_1 = 2.4$ and $\tau_2 = 3.4$ when $\Delta\tau = 1$ (Fig. 4B), and $\tau_1 = 1$ and $\tau_2 = 5$ when $\Delta\tau = 4$ (Fig. 4C). Then, we set $T_1 = T_2 = T$ and vary T between 3.44 and 5.71 for $\Delta\tau = 0$, between 3.45 and 5.72 for $\Delta\tau = 1$, and between 3.48 and 5.72 for $\Delta\tau = 4$. With this parameter setting, T_p is in the range between 21.6 and 26.4 hours as shown in Fig. 4A-C.

We run entrainment simulations for 120 LD cycles, and then calculate the variance of the peak value of m_1 with additional 30 LD cycles to determine whether the system is entrained to the cycle. As shown in Fig. 4D-G, if oscillations are entrained, the peak values of m_1 and m_2 are constant. Therefore, if the variance of m_1 peak values is less than 0.005, the oscillation is judged to be entrained to the LD cycle.

Numerical simulations of delay differential equations

To solve the delay differential equations (1) in the main text, we use the Euler method with time step of $\delta t = 0.005$ h. When we numerically solve the nondimensional equations (S2) and (S3), we set $\delta t = 0.002$. The codes were written in C language (S1 Supporting file).

Parameter values

We use the following standard set of parameter values in Eq. (1) for Figs 1-4 in the main text,

and S1-5 Figs in the supporting information: $\beta = \nu = 1$, $\alpha = \mu = 0.4$, $K = 1.8$, $\tau_1 = 1$, $T_1 = T_2 = 5$, and $n = 4$. In Fig. 4 and S9 Fig, the time delay parameters τ_i and T_i are changed as described in the above section. We use the duration of light signal $T_d = 1 \times T_p/24$ in Eq. (2) unless mentioned otherwise. In Figs. 2, 4 and S3, S4, S5D-K Figs, $\bar{\tau}_1 = \tau_1$ and $\bar{\tau}_2 = \tau_2$. In Fig. 3, $\bar{\tau}_1 = 1$ and $\bar{\tau}_2 = 1.5$.

In the nondimensional form Eq. (S2), we set a standard parameter set as: $\kappa_{12} = \kappa_{12} = \kappa_{22} = 1$, $b = 1$, $a = 1$, $f_1 = f_2 = 3.47$, $h_1 = h_2 = 1$, $n_1 = n_2 = 4$, $\tilde{\tau}_1 = \bar{\bar{\tau}}_1 = 0.4$, $\tilde{\tau}_2 = \bar{\bar{\tau}}_2 = 2$, and $\tilde{T}_1 = \tilde{T}_2 = 2$. We vary one of these parameters with all the other parameters fixed as the above standard values to analyze parameter dependence of phase responses in S6A-E, G, H Fig. In the nondimensional equation Eq. (S3), we set $n = 4$, $\tilde{\tau}_1 = \bar{\bar{\tau}}_1 = 0.4$, $\tilde{\tau}_2 = \bar{\bar{\tau}}_2 = 2$, and $\tilde{T}_1 = \tilde{T}_2 = 2$. $f = \nu\beta/(\alpha^2 K)$ and $h = \mu/\alpha$ are set as shown in S6I-K Fig.

For the analyses of phase responses with Eqs. (S2) and (S3), the nondimensional duration of light signal is set as $\tilde{T}_d = 0.4 \times T_p/9.6$. The nondimensional light-induced transcription rates are set as: $\tilde{\epsilon}_1 = 0.5$ and $\tilde{\epsilon}_2 = 0$ for only *P1* induction case, and $\tilde{\epsilon}_1 = 0$ and $\tilde{\epsilon}_2 = 0.5$ for only *P2* induction case.

Models for dual negative feedback loops including multiple states of mRNA and protein

In the main text, we used delay differential equations to model dual negative feedback loops (NFLs) of *Per1* and *Per2*. As a complementary approach, here, we adopt systems of ordinary differentiation equations (ODEs) and examine whether the peak time difference between two repressors causes phase responses as in the model based on delay differential equations. To describe time delays in a NFL, we include multiple biochemical reaction steps by using sets of ODEs.

We first analyze a model for a single NFL to study how the period and amplitude of oscillation and phase responses, depend on the number of reaction steps and their time constant. Then, we consider dual NFLs that model regulation of the two *Per* genes. We will confirm that the time difference in expression peaks between two repressors also causes complementary phase responses in the ODE-based model.

Single NFL model

We consider multiple states of repressor mRNA and protein that result in effective delays in feedback regulation [1]. For instance, transcribed mRNA includes both introns and exons.

After splicing these introns, mRNA may be subject to some additional modification. Then, mRNA is transported from nucleus to cytoplasm for translation. Similarly, after translation of mRNA, a repressor protein may be phosphorylated and transported from cytoplasm to nucleus. The protein may form complex with other clock proteins. These different states of mRNA and protein can be described with separate variables in ODEs. Previous mathematical models for mammalian circadian clocks considered multiple phosphorylation states of PER proteins [2] and various complex formation of circadian clock proteins [3-7].

For simplicity, we model state transition of mRNA and protein by linear chains of reactions with time constants (S7A Fig). Let m_{1i} (i = 1, 2, .., u) be the levels of mRNA in state i. m_{11} and m_{1u} represent the levels of nascent mRNA in the nucleus, and fully matured mRNA in cytoplasm available for translation, respectively. m_{1i} (i = 2, .., u−1) represents the levels of all the other intermediatory states of mRNA. We describe the time evolution of m_{1i} using a series of ODEs:

$$\frac{dm_{11}(t)}{dt} = \frac{\beta}{1 + (p_{1r}(t)/K)^n} + \gamma(t) - \eta \cdot m_{11}(t), \qquad (S4a)$$

$$\frac{dm_{1i}(t)}{dt} = \eta \cdot \left(m_{1i-1}(t) - m_{1i}(t)\right), \qquad (i = 2, 3, \ldots, u - 1) \qquad (S4b)$$

$$\frac{dm_{1u}(t)}{dt} = \eta \cdot m_{1u-1}(t) - \alpha \cdot m_{1u}(t), \qquad (S4c)$$

where β is the maximum light-independent transcription rate, K is the dissociation constant of the repressor protein to its own promoter, n is the Hill coefficient and α is the degradation rate of the fully matured mRNA. η is the time constant of state transition from i to i + 1. For simplicity, we consider the same time constant for each transition. p_{1r} is the levels of functional repressor protein at nucleus as described below. $\gamma(t)$ represents light-induced transcription rate. We use the rectangular function Eq. (2) to model $\gamma(t)$ as in the main text.

Similarly, let p_{1j} (j = 1, 2, .., r) be the levels of protein in state j (S7A Fig). We describe time evolution of p_{1j} as:

$$\frac{dp_{11}(t)}{dt} = \nu \cdot m_{1u}(t) - \lambda \cdot p_{11}(t), \qquad (S4d)$$

$$\frac{dp_{1j}(t)}{dt} = \lambda \cdot \left(p_{1j-1}(t) - p_{1j}(t)\right), \qquad (j = 2, 3, \ldots, r - 1) \qquad (S4e)$$

$$\frac{dp_{1r}(t)}{dt} = \lambda \cdot p_{1r-1}(t) - \mu \cdot p_{1r}(t), \qquad (S4f)$$

where ν is the translation rate, λ is the time constant of state transition from *j* to *j* + 1, and μ is the degradation rate of the functional protein p_{1r}.

We first study the dependence of the period and amplitude on the time constants η (S7B, C Fig) and the state number of mRNA u (S7E, F Fig), because these two parameters are relevant to the peak time difference between repressor mRNAs when we consider dual NFLs below. We fix $\lambda = 0.6$ and $r = 4$ for the repressor protein in the following analysis. Values of other reaction parameters in Eq. (S4) are: $\beta = \nu = 1.5$, $K = 1.8$, $n = 4$, and $\alpha = \mu = 0.6$. For the light-induced transcription rate $\gamma(t)$ in Eq. (2), we use $\epsilon = 1$ and $T_d = 1 \times T_p/24$. For numerical integration, we use the 4th order Runge-Kutta method with the time step of $\delta t = 0.01$ h.

S7B, C Fig shows the dependence of period and amplitude of p_{1r} on the time constant η with *u* = 4. As η increases, the period of oscillation decreases and m_{1u} peaks earlier (S7B, C Fig). In contrast, the amplitude of p_{1r} increases with η (S7C Fig), indicating that slow state transition is detrimental to amplitude. Despite the difference in period and amplitude, we obtain similar phase response curves (PRCs) for $\eta = 0.35$ and 0.7 when plotting them in circadian time (CT; S7D Fig). Due to the period difference, PRC with $\eta = 0.7$ is shifted right from that with $\eta = 0.35$. In both cases, however, PRCs of this single NFL include advance and delay zones.

Next, we analyze the dependence of these rhythm properties on the state number of mRNA u (S7E-G Fig). In S7E-G Fig, we fix $\eta = 0.5$. Unlike the dependence on η, both the period and amplitude increase with *u* (S7E, F Fig). Although the amplitude of PRC slightly decreases with *u*, PRC shape only weakly depends on it (S7G Fig). Thus, we confirm that PRCs of the single NFL described by the set of ODEs include both advance and delay zones regardless of the length of time delays in mRNA synthesis.

Dual NFL model

Next, we consider two interacting NFLs and describe them with the system of ODEs as in the previous section (S8A Fig). m_{1i} represents the levels of *P1* mRNA in state *i* (*i* = 1, 2, .., *u*). Similarly, m_{2j} represents the levels of *P2* mRNA in state *j* (*j* = 1, 2, .., *w*). To generate 4-hour peak time difference between m_{1u} and m_{2w}, we assume that the state number *w* for *P2* mRNA can be larger than *u* for *P1* mRNA. In addition, variables p_{1i} and p_{2i} represent the *i*th states of P1 and P2 proteins, respectively (*i* = 1, 2, .., *r*). For simplicity, we consider the same state number *r* for both P1 and P2 proteins. We describe time evolution of these

variables as:

$$\frac{dm_{\#1}(t)}{dt} = \frac{\beta}{1 + (p_{1r}(t)/K)^n + (p_{2r}(t)/K)^n} + \gamma_{\#}(t) - \eta_{\#} \cdot m_{\#1}(t), \qquad (S5a)$$

(for # = 1 and 2)

$$\frac{dm_{\#i}(t)}{dt} = \eta_{\#} \cdot \left(m_{\#i-1}(t) - m_{\#i}(t)\right), \qquad (S5b)$$

$(i = 2, 3, .., u - 1$ for # = 1 and $i = 2, 3, .., w - 1$ for # = 2)

$$\frac{dm_{1u}(t)}{dt} = \eta_1 \cdot m_{1u-1}(t) - \alpha \cdot m_{1u}(t), \qquad (S5c)$$

$$\frac{dm_{2w}(t)}{dt} = \eta_2 \cdot m_{2w-1}(t) - \alpha \cdot m_{2w}(t), \qquad (S5d)$$

$$\frac{dp_{11}(t)}{dt} = \nu \cdot m_{1u}(t) - \lambda \cdot p_{11}(t), \qquad (S5e)$$

$$\frac{dp_{21}(t)}{dt} = \nu \cdot m_{2w}(t) - \lambda \cdot p_{21}(t), \qquad (S5f)$$

$$\frac{dp_{\#j}(t)}{dt} = \lambda \cdot \left(p_{\#j-1}(t) - p_{\#j}(t)\right), \qquad (S5g)$$

$(j = 2, 3, .., r - 1$ for # = 1 and 2)

$$\frac{dp_{\#r}(t)}{dt} = \lambda \cdot p_{\#r-1}(t) - \mu \cdot p_{\#r}(t), \qquad \text{(for \# = 1 and 2)} \qquad (S5h)$$

where we also assume the different time constants for state transition η_1 and η_2 in *P1* and *P2* mRNAs (S8A Fig). The notations of all the other parameters are the same in the single NFL model introduced in the previous section. We use the following parameter values for the analysis described below: $\beta = \nu = 1.5$, $K = 1.8$, $n = 4$, $\alpha = \mu = 0.6$, $u = 3$, $\eta_1 = 0.6$, $\lambda = 0.6$ and $r = 4$. For the light-induced transcription rates $\gamma_1(t)$ and $\gamma_2(t)$ in Eq. (S5a), we use $(\epsilon_1, \epsilon_2) = (0.7, 0)$ for only *P1* mRNA induction, $(\epsilon_1, \epsilon_2) = (0, 0.7)$ for only *P2* mRNA induction, and $(\epsilon_1, \epsilon_2) = (0.7, 0.7)$ for simultaneous induction. The light duration is $T_d = 1 \times T_p/24$.

We first analyze the dependence of peak time difference between m_{1u} and m_{2w} on state number w and the ratio of time constants η_2/η_1 (S8B Fig). We fix u = 3 and $\eta_1 = 0.6$ and change the value of w and η_2 in S8B Fig. The peak time difference becomes large with small values of η_2/η_1 for all state numbers w examined. However, decreasing η_2/η_1 also compromises the amplitude of p_{2r} (S8B, C Fig). For example, although the system with $w = u = 3$ can generate peak time difference between m_{1u} and m_{2w} nearly 4 hours (S8B Fig), the amplitude of p_{2r} is almost 0 (S8C Fig). As the difference in state number $w - u$

becomes large, larger values of η_2/η_1 attain 4-hour peak time difference with considerable amplitude (S8B-E Fig). Therefore, we decide to examine PRCs with $w = 4$ and 5.

We observe complementary phase responses in this description of dual NFLs (S8F, G Fig). Namely, light induction of *P1* mRNA contributes mainly to phase advance, and that of *P2* mRNA contributes to phase delay. As in the delay differential equation model in the main text, we observe the additivity of PRCs in this ODE-based model (gray lines in S8F, G Fig). For w = 4 in S8F Fig., the peak value of PRC with light induction of only *P1* mRNA ($\varepsilon_1 = 0.7$, $\varepsilon_2 = 0$) is larger than the trough value of PRC with light induction of only *P2* mRNA ($\varepsilon_1 = 0$, $\varepsilon_2 = 0.7$). This is probably due to the difference in amplitudes of p_{1r} and p_{2r} caused by the difference in time constants.

Supplementary References

1. Novak B, Tyson JJ. Design principles of biochemical oscillators. Nat Rev Mol Cell Biol. 2008;9(12):981-91. doi: 10.1038/nrm2530. PMID: 18971947.
2. Zhou M, Kim JK, Eng GW, Forger DB, Virshup DM. A Period2 Phosphoswitch Regulates and Temperature Compensates Circadian Period. Mol Cell. 2015;60(1):77-88. doi: 10.1016/j.molcel.2015.08.022. PMID: 26431025.
3. Kim JK, Forger DB. A mechanism for robust circadian timekeeping via stoichiometric balance. Mol Syst Biol. 2012;8:630. doi: 10.1038/msb.2012.62. PMID: 23212247.
4. Forger DB, Peskin CS. A detailed predictive model of the mammalian circadian clock. Proc Natl Acad Sci U S A. 2003;100(25):14806-11. doi: 10.1073/pnas.2036281100. PMID: 14657377.
5. Leloup JC, Goldbeter A. Toward a detailed computational model for the mammalian circadian clock. Proc Natl Acad Sci U S A. 2003;100(12):7051-6. doi: 10.1073/pnas.1132112100. PMID: 12775757.
6. Mirsky HP, Liu AC, Welsh DK, Kay SA, Doyle FJ, 3rd. A model of the cell-autonomous mammalian circadian clock. Proc Natl Acad Sci U S A. 2009;106(27):11107-12. doi: 10.1073/pnas.0904837106. PMID: 19549830.
7. Relogio A, Westermark PO, Wallach T, Schellenberg K, Kramer A, Herzel H. Tuning the mammalian circadian clock: robust synergy of two loops. PLoS Comput Biol. 2011;7(12):e1002309. doi: 10.1371/journal.pcbi.1002309. PMID: 22194677.
