# Supplementary figures and images for: Complementary phase responses via functional differentiation of dual negative feedback loops
Source: PLoS Comput Biol. 2021 Mar 8;17(3):e1008774. doi: 10.1371/journal.pcbi.1008774 (PMC7971863; doi:10.1371/journal.pcbi.1008774)

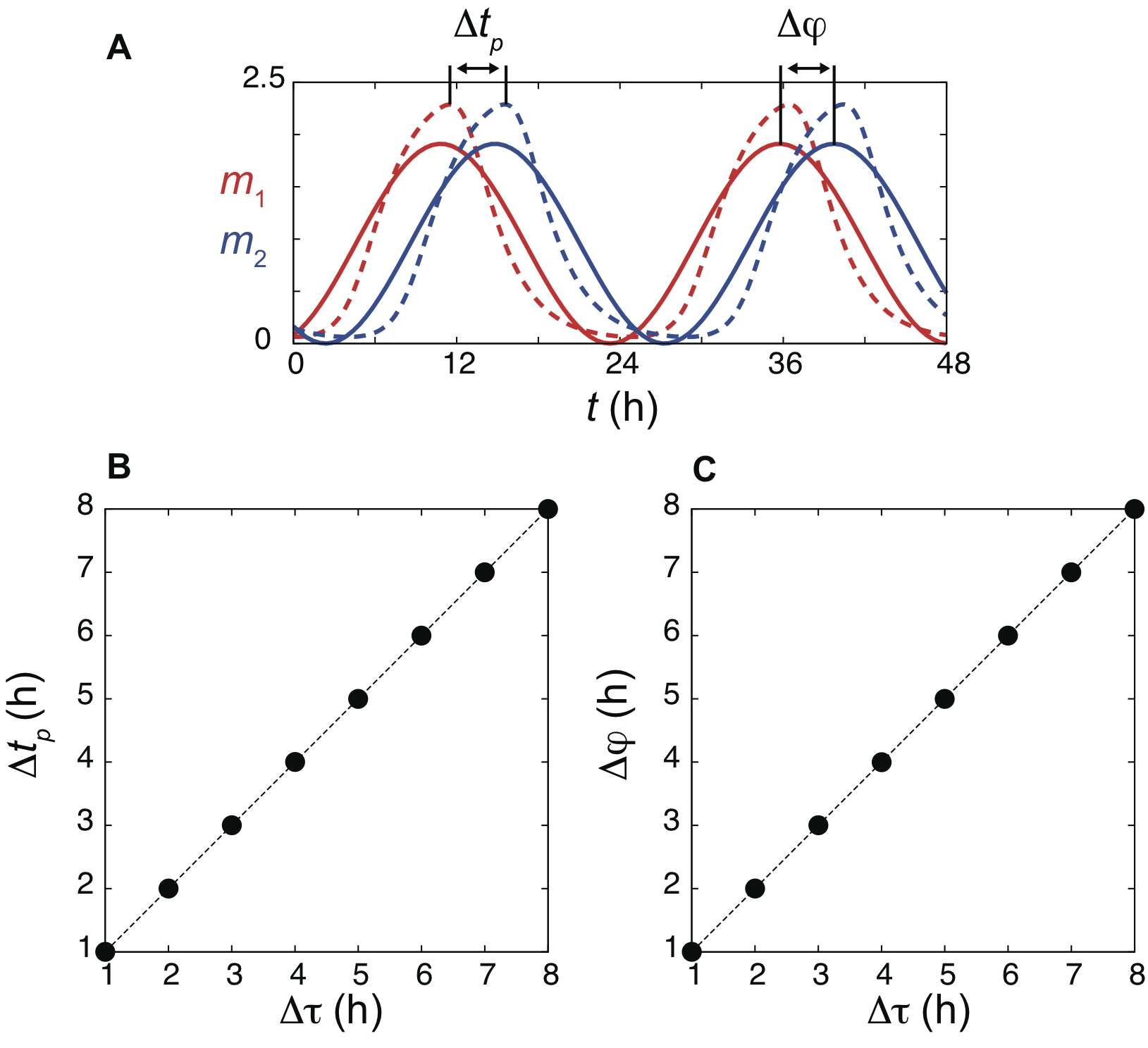

Supplement: S1 Fig — (A) Time series of P1 (m1: red dotted) and P2 (m2: blue dotted) mRNAs. The solid red and blue lines indicate the first Fourier modes of m1 and m2, respectively. Δtp is the peak time difference between m1 and m2. Δφ is the phase difference between the first Fourier modes. (B) Correlation between Δτ and Δtp. (C) Correlation between Δτ and Δφ. In (B) and (C), dotted diagonal lines indicate y = x. (TIFF) [file pcbi.1008774.s003.tiff]

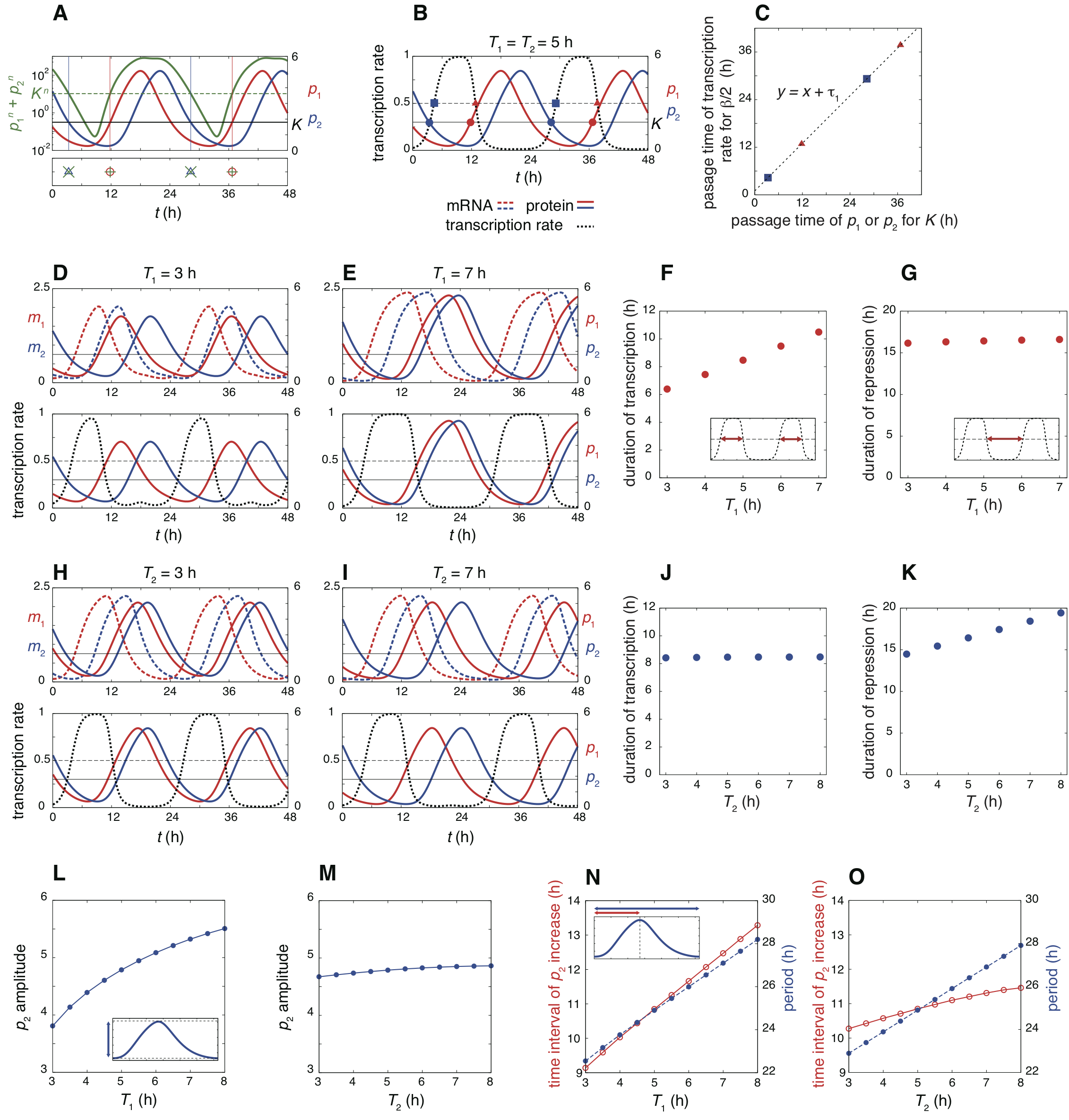

Supplement: S2 Fig — (A) (Top) Timeseries of p1 (red solid), p2 (blue solid), and p1n + p2n (green solid). The black horizontal line indicates the dissociation constant K. Green horizontal line indicates Kn. Vertical lines indicate the time at which p1n + p2n becomes larger (red) or smaller (blue) than Kn. Log scale in left y-axis for p1n + p2n and linear scale in right y-axis for p1 and p2. (Bottom) Green marks × and + indicate time at which p1n + p2n becomes smaller and larger than Kn, respectively. Red open circles indicate time at which p1 becomes larger than K. Blue open triangles indicate time at which p2 becomes smaller than K. (B) Timeseries of the light-independent transcription rate of P1 mRNA in Eq (1a) (black dotted), p1 (red solid) and p2 (blue solid). The solid horizontal line indicates the dissociation constant K. The dotted horizontal line indicates β/2 where β = 1. Red and blue circles indicate passage time of p1 and p2 for K, respectively. Red triangles and blue squares indicate the times at which light-independent transcription rate decreases to and increases to β/2, respectively. These passage times are plotted in (C). (C) Passage time of light-independent transcription rate of P1 mRNA in Eq (1a) for β/2 as a function of the passage time of p1 or p2 for K. Blue squares indicate the relation between the time at which the transcription rate increases to β/2 and the one at which p2 becomes smaller than K. Red triangles indicate the relation between the time at which the transcription rate decreases to β/2 and the one at which p1 becomes larger than K. The dotted line indicates y = x + τ1. (D), (E), (H), (I) Timeseries of (top) mRNAs mi and proteins pi, and (bottom) light-independent transcription rate for the different values of time delays in translation Ti. (D) T1 = 3 h and (E) T1 = 7 h with T2 = 5 h. (H) T2 = 3 h and (I) T2 = 7 h with T1 = 5 h. In the bottom panels, timeseries of p1 and p2 are also plotted. Solid horizontal lines indicate K. Dotted horizontal lines i [file pcbi.1008774.s004.tiff]

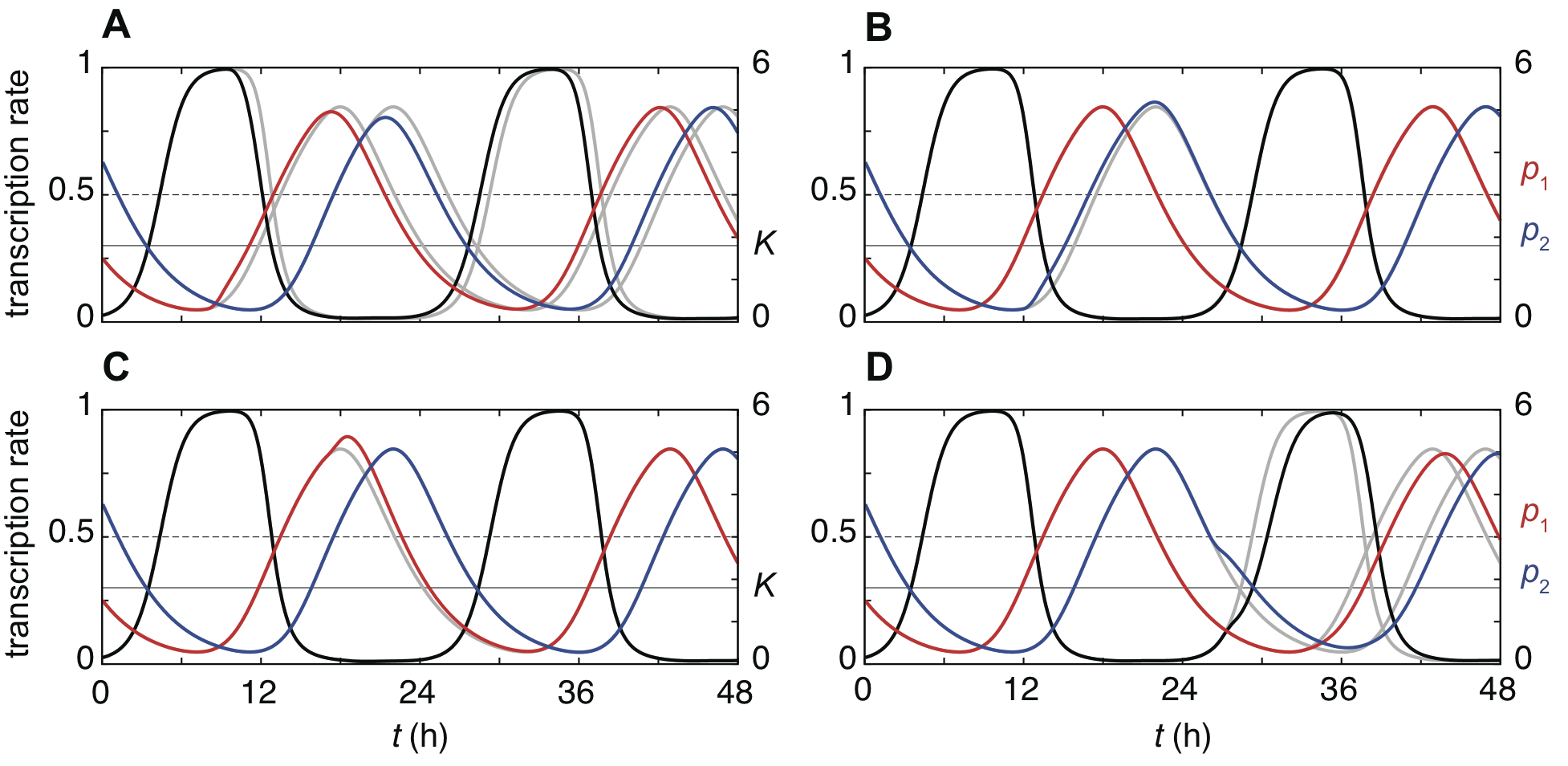

Supplement: S3 Fig — (A)-(D) Timeseries of light-independent transcription rate of P1 mRNA in Eq (1a) (black), levels of P1 (red) and P2 (blue) proteins in the presence of a light signal. Each panel corresponds to Fig 2G–2J in the main text. A light signal is administered at (A), (B) tl = 2 h, (C) tl = 11 h and (D) tl = 16 h. In (A) and (C), only P1 mRNA is induced by the light signal. In (B) and (D), only P2 mRNA is induced by the light signal. Gray lines indicate timeseries in the absence of a light signal. Solid horizontal lines indicate the dissociation constant K. Dotted horizontal lines indicate β/2 with β = 1. (TIFF) [file pcbi.1008774.s005.tiff]

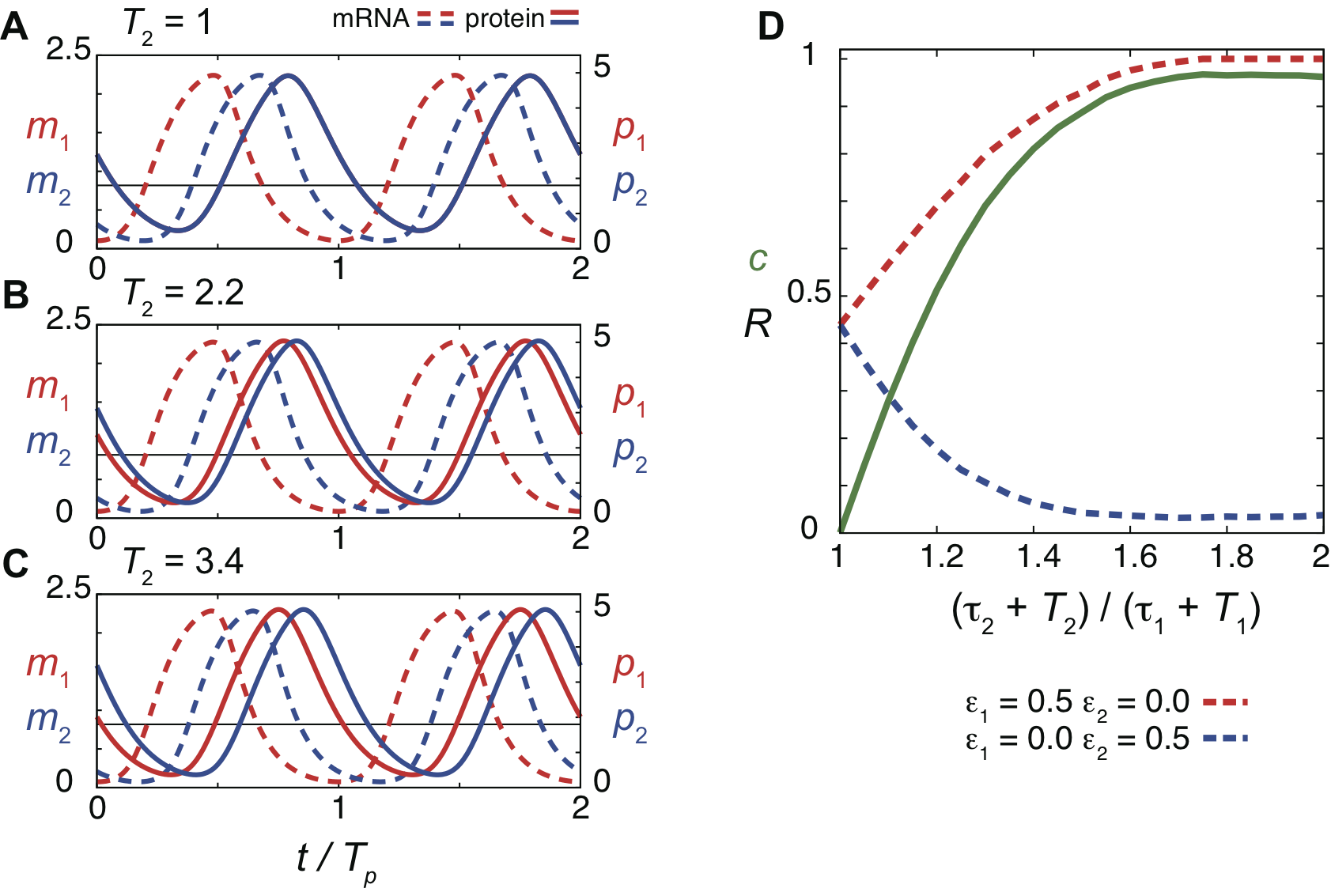

Supplement: S4 Fig — (A)-(C) Time series of mRNAs and proteins for (A) T2 = 1 h, (B) T2 = 2.2 h and (C) T2 = 3.4 h. Horizontal lines indicate the dissociation constant K in Eq (1a). For better comparison, time is normalized by the autonomous period Tp. (D) Dependence of the area fraction R (red and blue dotted) and index c (green solid) on the ratio of total time delays in each negative feedback loop. T2 changes from 1 to 7 hours with all the other parameters fixed. In all panels, τ1 = 1 h, τ2 = 5 h and T1 = 5 h. (TIFF) [file pcbi.1008774.s006.tiff]

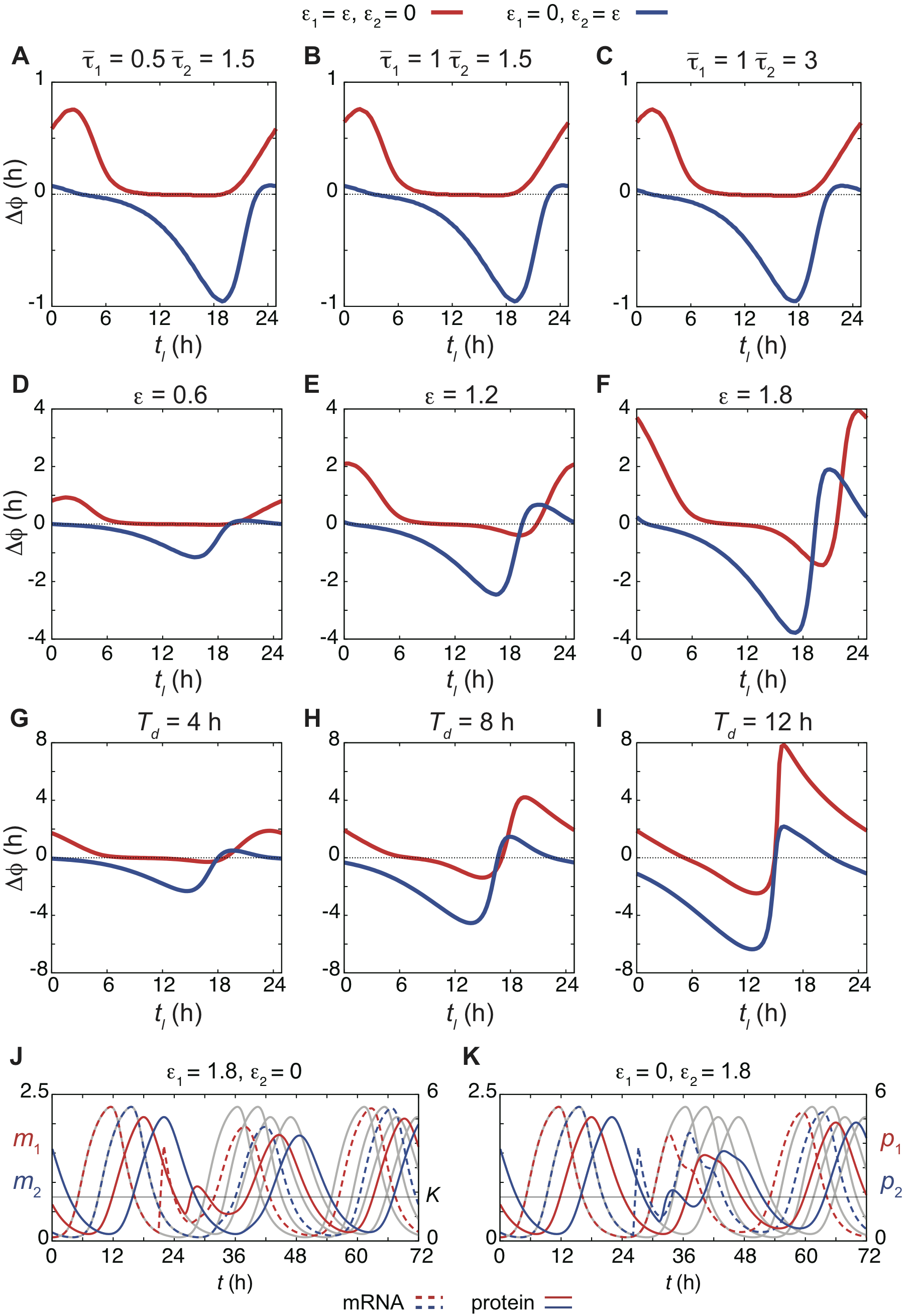

Supplement: S5 Fig — (A)-(I) Dependence of PRCs on (A)-(C) time delays τ¯i, (D)-(F) light induced transcription rate ϵ and (G)-(I) light duration Td in Eqs (1a) and (2). The red lines indicate PRCs with ϵ1 = ϵ and ϵ2 = 0. The blue lines indicate PRCs with ϵ1 = 0 and ϵ2 = ϵ. In (A)-(C), ϵ = 0.5 and Td = 1 h. In (D)-(F), τ¯1=1 h, τ¯2=5 h and Td = 1 h. In (G)-(H), ϵ=0.5,τ¯1=1 h, and τ¯2=5 h. (J), (K) Time series of mRNAs and proteins in the presence of a light signal (red and blue lines). Light induction of only (J) P1 or (K) P2 mRNA. Horizontal lines indicate the dissociation constant K in Eq (1a). Gray lines indicate time series in the absence of a light signal. Td = 1 h. (TIFF) [file pcbi.1008774.s007.tiff]

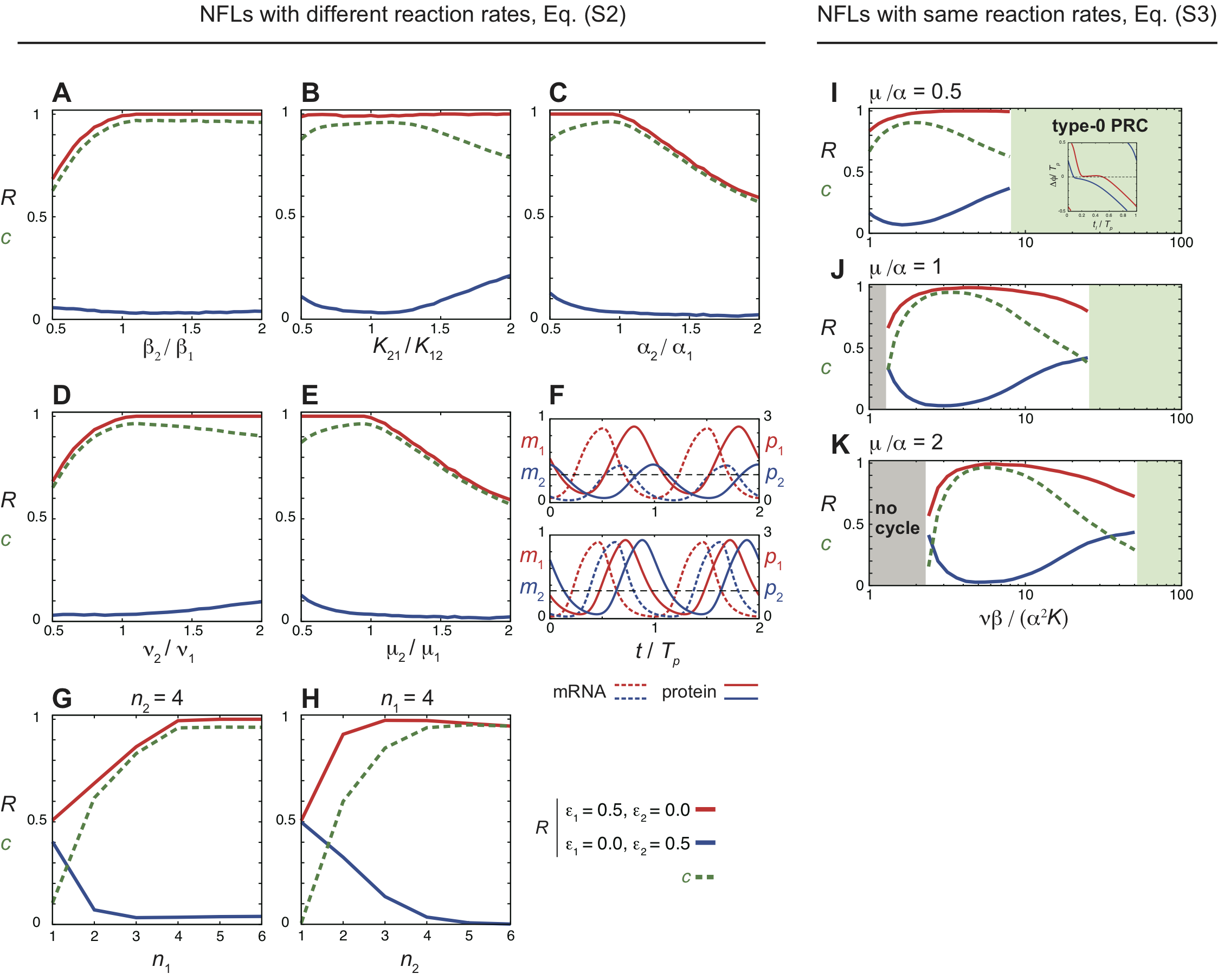

Supplement: S6 Fig — (A)-(E) Dependence of the area fraction R and complementarity index c on ratios of nondimensional reaction parameters. Results of the two NFLs in an extended model Eq. (S2) in S1 Text. Dependence on (A) the ratio of light-independent transcription rates b = β2/β1, (B) dissociation constants κ21/κ12 = K2/K1, (C) degradation rates of mRNAs a = α2/α1, (D) translation rates f2/f1 = ν2/ν1, and (E) degradation rates of proteins h2/h1 = μ2/μ1. In (B), we fix κ12 =1 and change κ21 in Eq. (S2). In (D), f1 = 3.47 and we change f2. In (E), h1 = 1 and we change h2. (F) Timeseries of mRNAs and proteins with (top) b = β2/β1 = 0.5 and (bottom) b = β2/β1 = 1 in the absence of light signals. Dotted horizontal lines indicate the value of the dissociation constant κ = κij (i, j = 1, 2). (G), (H) Dependence of R and c on the Hill coefficients (G) n1 and (H) n2 in Eq. (S2). (I)-(K) Dependence of R and c on the nondimensional parameter f = νβ/(α2K) for different values of h = μ/α in Eq. (S3) in S1 Text. (I) μ/α = 0.5, (J) μ/α = 1, and (K) μ/α = 2. Gray shades indicate the parameter regions where a steady state is stable and there is no limit cycle solution. Green shades indicate the parameter regions where the shape of PRCs is discontinuous type-0 (inset in (I)). See S1 Text for derivation of a nondimensional form of Eq (1) and values of parameters. (TIFF) [file pcbi.1008774.s008.tiff]

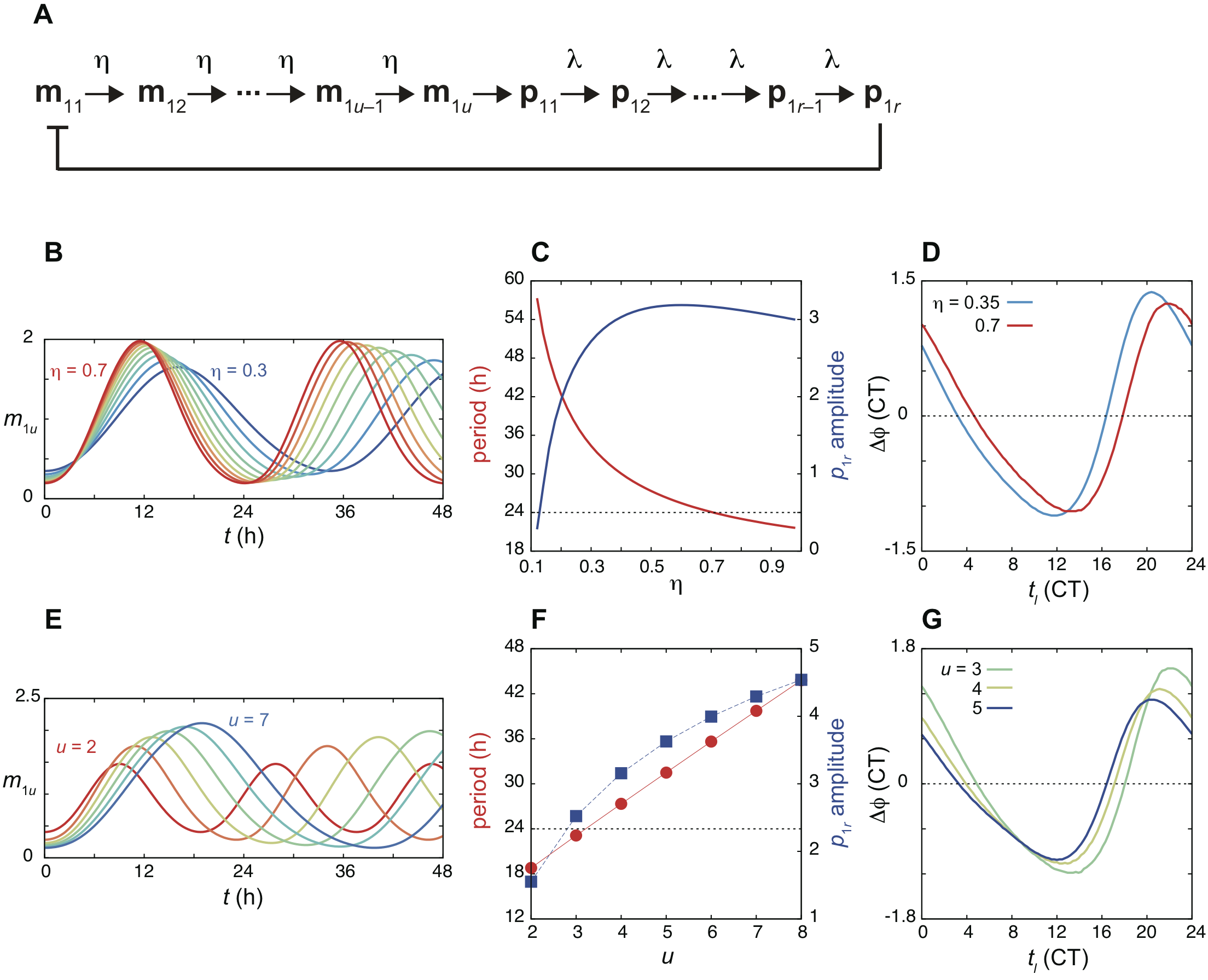

Supplement: S7 Fig — (A) Schematic of a single negative feedback loop including a linear chain of mRNA and protein state transitions. η is the time constant of mRNA state transition and λ is that of protein state transition. The functional protein p1r represses its own transcription. (B) Time series of the functional mRNA m1u available for translation for different values of η. The value of η changes from 0.3 (blue) to 0.7 (red) with the step size of 0.05. (C) Dependence of period and amplitude of oscillation on η. The amplitude of functional protein p1r is plotted. (D) Phase response curves (PRCs) for the light induction of m11 with two different values of η. (E) Time series of the functional mRNA m1u for different state number u. The value of u changes from 2 (red) to 7 (blue). (F) Dependence of period (red circles) and amplitude (blue squares) of oscillation on u. (G) PRCs for the light induction of m11 with different values of u. Dotted horizontal lines in (C) and (F) indicate period of 24 hours as a reference. In (D) and (G), phase shift Δϕ and administration time tl are indicated in the unit of circadian time (CT) for better comparison. The values of other parameters are listed in S1 Text. (TIFF) [file pcbi.1008774.s009.tiff]

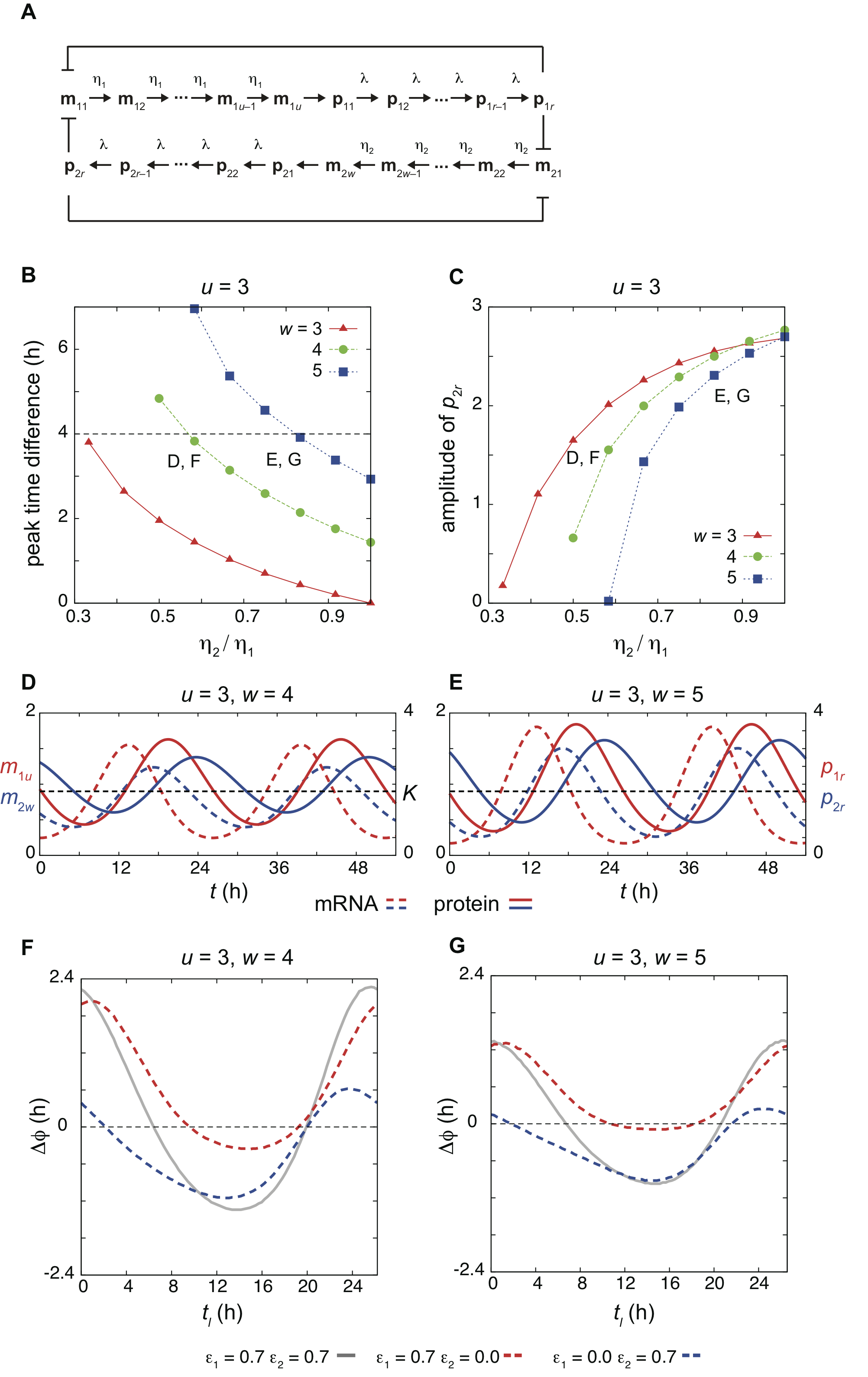

Supplement: S8 Fig — (A) Schematic of dual negative feedback loops including linear chains of mRNA and protein state transitions. η1 is the time constant of P1 mRNA state transition and η2 is that of P2 mRNA state transition. λ is the time constant of protein state transition. Functional proteins p1r and p2r repress their own and opponent’s transcription. (B) Dependence of peak time difference between m1u and m2w on the ratio of time constants of state transition η2/η1. Results for different values of state number of P2 mRNA w are shown. Dotted horizontal line indicates 4-hour peak time difference as a reference. (C) Dependence of the amplitude of p2r on η2/η1 for different values of w. (D), (E) Time series of functional mRNAs and proteins for (D) w = 4 and η2/η1 = 0.583, and (E) w = 5 and η2/η1 = 0.833. Dotted horizontal lines indicate the dissociation constant K. (F), (G) Phase response curves to light signals for (F) w = 4 and η2/η1 = 0.583, and (G) w = 5 and η2/η1 = 0.833. In (B)-(G), u = 3. The values of other parameters are listed in S1 Text. (TIFF) [file pcbi.1008774.s010.tiff]

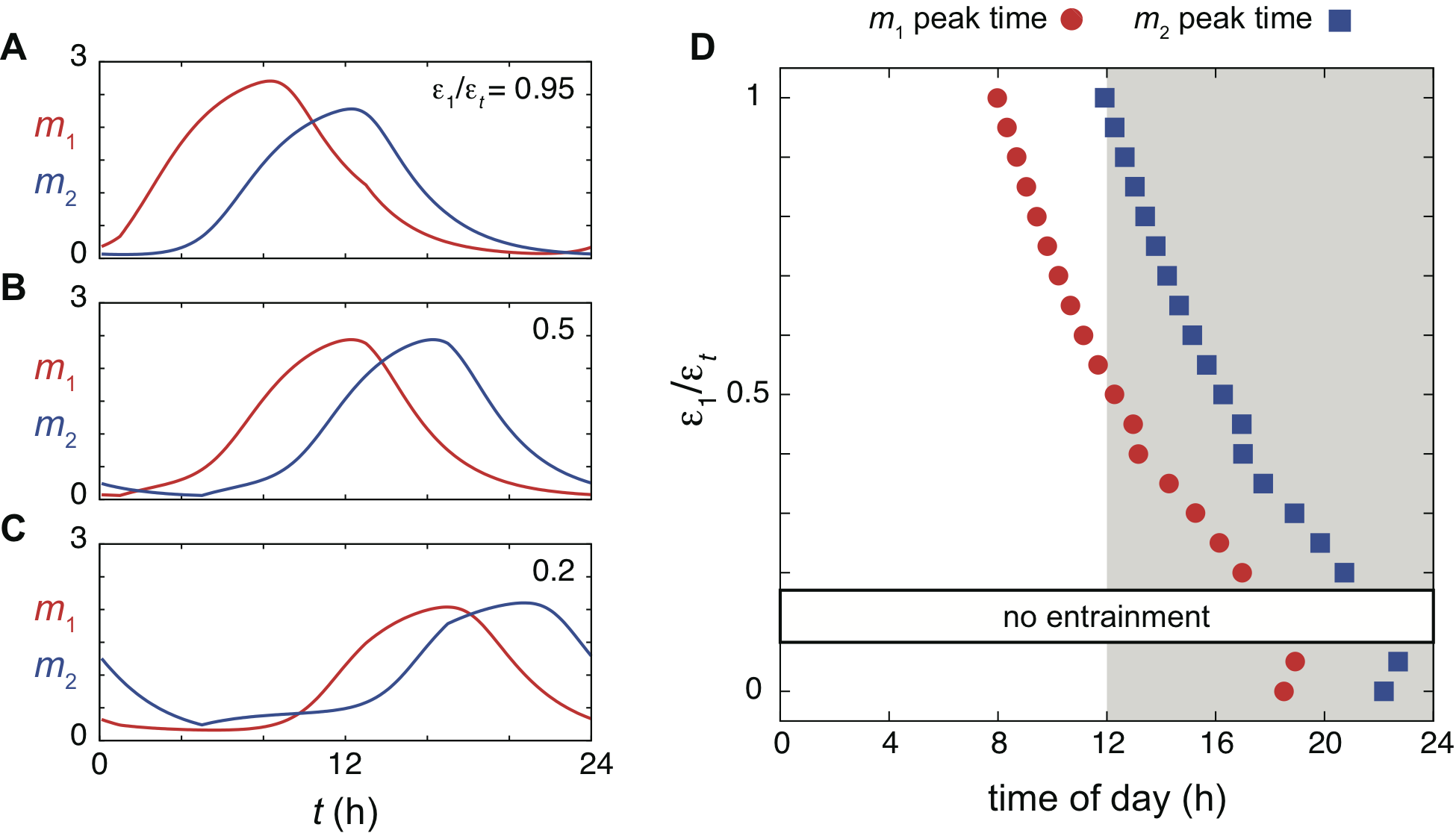

Supplement: S9 Fig — (A)-(C) Time series of P1 (red) and P2 (blue) mRNAs in the presence of a 12:12 light-dark (LD) cycle. The ratio of light-induced transcription rates is (A) ϵ1/ϵt = 0.95, (B) ϵ1/ϵt = 0.5 and (C) ϵ1/ϵt = 0.2. Entrained rhythms of m1 and m2 are plotted. (D) Dependence of peak time of P1 and P2 mRNAs on ϵ1/ϵt. The gray shade region indicates time interval where light signal is off. The autonomous period is 24.51 hours with T1 = T2 = 4.82 h in Eq (1b). (TIFF) [file pcbi.1008774.s011.tiff]
